# Supplementary material for: The Role of Vitamin D in Parkinson’s Disease: Evidence from Serum Concentrations, Supplementation, and VDR Gene Polymorphisms
Source: NeuroSci. 2025 Dec 16;6(4):130. doi: 10.3390/neurosci6040130 (PMC12735780; doi:10.3390/neurosci6040130)
Supplement: Supplementary file 1 [file neurosci-06-00130-s001.zip › neurosci-3909137-supplementary.pdf]

NeuroSci

Supplementary Material

Content

Table S1. PRISMA 2020 - Checklist.
Table S2. FreeText and MeSH search terms in the US National Library of Medicine.
Table S3. Chromosome 12 SNPs Associated with the Vitamin D Receptor.
Table S4. VitD assay methods.
Table S5. Quality assessment of observational studies of vitamin D levels based on Newcastle-Ottawa Scale.
Table S6. Quality assessment of clinical trial studies based on robvis.
Table S7. Quality assessment of observational studies of genetic polymorphism based on Newcastle-Ottawa Scale.
Table S8. Serum 25(OH)D levels in PD versus control.
Table S9. 25(OH)D insufficiency in PD versus control.
Table S10. 25(OH)D deficiency in PD versus control.
Table S11. VitD and PD incidence.
Table S12. VitD supplementation versus placebo in PD.
Table S13. Comparison between alleles and genotypes of VitD SNPs and Parkinson's disease.
Table S14. Effects of Vitamin D in In Vitro Models of Parkinson's Disease.
Table S15. Effects of Vitamin D in In Vivo Models of Parkinson's Disease.
Table S16. Amounts of Vitamin D and Energy per Standard Portion.
Table S17. Clinical trials assessing the effect of VitD on PD.

TABLE S1

**Table S1. PRISMA 2020 - Checklist.**			
**Section and Topic**	**Item #**	**Checklist item**	**Location where item is reported**
**TITLE**			
Title	1	Identify the report as a systematic review.	
**ABSTRACT**			
Abstract	2	See the PRISMA 2020 for Abstracts checklist.	
**INTRODUCTION**			
Rationale	3	Describe the rationale for the review in the context of existing knowledge.	
Objectives	4	Provide an explicit statement of the objective(s) or question(s) the review addresses.	
**METHODS**			
Eligibility criteria	5	Specify the inclusion and exclusion criteria for the review and how studies were grouped for the syntheses.	
Information sources	6	Specify all databases, registers, websites, organisations, reference lists and other sources searched or consulted to identify studies. Specify the date when each source was last searched or consulted.	
Search strategy	7	Present the full search strategies for all databases, registers and websites, including any filters and limits used.	
Selection process	8	Specify the methods used to decide whether a study met the inclusion criteria of the review, including how many reviewers screened each record and each report retrieved, whether they worked independently, and if applicable, details of automation tools used in the process.	
Data collection process	9	Specify the methods used to collect data from reports, including how many reviewers collected data from each report, whether they worked independently, any processes for obtaining or confirming data from study investigators, and if applicable, details of automation tools used in the process.	
Data items	10a	List and define all outcomes for which data were sought. Specify whether all results that were compatible with each outcome domain in each study were sought (e.g. for all measures, time points, analyses), and if not, the methods used to decide which results to collect.	
	10b	List and define all other variables for which data were sought (e.g. participant and intervention characteristics, funding sources). Describe any assumptions made about any missing or unclear information.	
Study risk of bias assessment	11	Specify the methods used to assess risk of bias in the included studies, including details of the tool(s) used, how many reviewers assessed each study and whether they worked independently, and if applicable, details of automation tools used in the process.	
Effect measures	12	Specify for each outcome the effect measure(s) (e.g. risk ratio, mean difference) used in the synthesis or presentation of results.	
Synthesis methods	13a	Describe the processes used to decide which studies were eligible for each synthesis (e.g. tabulating the study intervention characteristics and comparing against the planned groups for each synthesis (item #5)).	

https://doi.org/10.3390/neurosci6040130

	13b	Describe any methods required to prepare the data for presentation or synthesis, such as handling of missing summary statistics, or data conversions.	
	13c	Describe any methods used to tabulate or visually display results of individual studies and syntheses.	
	13d	Describe any methods used to synthesize results and provide a rationale for the choice(s). If meta-analysis was performed, describe the model(s), method(s) to identify the presence and extent of statistical heterogeneity, and software package(s) used.	
	13e	Describe any methods used to explore possible causes of heterogeneity among study results (e.g. subgroup analysis, meta-regression).	
	13f	Describe any sensitivity analyses conducted to assess robustness of the synthesized results.	
Reporting bias assessment	14	Describe any methods used to assess risk of bias due to missing results in a synthesis (arising from reporting biases).	
Certainty assessment	15	Describe any methods used to assess certainty (or confidence) in the body of evidence for an outcome.	
**RESULTS**			
Study selection	16a	Describe the results of the search and selection process, from the number of records identified in the search to the number of studies included in the review, ideally using a flow diagram.	
	16b	Cite studies that might appear to meet the inclusion criteria, but which were excluded, and explain why they were excluded.	
Study characteristics	17	Cite each included study and present its characteristics.	
Risk of bias in studies	18	Present assessments of risk of bias for each included study.	
Results of individual studies	19	For all outcomes, present, for each study: (a) summary statistics for each group (where appropriate) and (b) an effect estimate and its precision (e.g. confidence/credible interval), ideally using structured tables or plots.	
Results of syntheses	20a	For each synthesis, briefly summarise the characteristics and risk of bias among contributing studies.	
	20b	Present results of all statistical syntheses conducted. If meta-analysis was done, present for each the summary estimate and its precision (e.g. confidence/credible interval) and measures of statistical heterogeneity. If comparing groups, describe the direction of the effect.	
	20c	Present results of all investigations of possible causes of heterogeneity among study results.	

https://doi.org/10.3390/neurosci6040130

	20d	Present results of all sensitivity analyses conducted to assess the robustness of the synthesized results.	
Reporting biases	21	Present assessments of risk of bias due to missing results (arising from reporting biases) for each synthesis assessed.	
Certainty of evidence	22	Present assessments of certainty (or confidence) in the body of evidence for each outcome assessed.	
**DISCUSSION**			
Discussion	23a	Provide a general interpretation of the results in the context of other evidence.	
	23b	Discuss any limitations of the evidence included in the review.	
	23c	Discuss any limitations of the review processes used.	
	23d	Discuss implications of the results for practice, policy, and future research.	
**OTHER INFORMATION**			
Registration and protocol	24a	Provide registration information for the review, including register name and registration number, or state that the review was not registered.	
	24b	Indicate where the review protocol can be accessed, or state that a protocol was not prepared.	
	24c	Describe and explain any amendments to information provided at registration or in the protocol.	
Support	25	Describe sources of financial or non-financial support for the review, and the role of the funders or sponsors in the review.	
Competing interests	26	Declare any competing interests of review authors.	
Availability of data, code and other materials	27	Report which of the following are publicly available and where they can be found: template data collection forms; data extracted from included studies; data used for all analyses; analytic code; any other materials used in the review.	

From: Page MJ, McKenzie JE, Bossuyt PM, Boutron I, Hoffmann TC, Mulrow CD, et al. The PRISMA 2020 statement: an updated guideline for reporting systematic reviews. BMJ 2021;372:n71. doi: 10.1136/bmj.n71. This work is licensed under CC BY 4.0. To view a copy of this license, visit https://creativecommons.org/licenses/by/4.0/

TABLE S2

Table S2. FreeText and MeSH search terms in the US National Library of Medicine.	
PubMed/ Medline	
Query	Results
("vitamin d"[Supplementary Concept] OR "vitamin d"[All Fields] OR "ergocalciferols"[Supplementary Concept] OR "ergocalciferols"[All Fields] OR "vitamin d"[MeSH Terms] OR "ergocalciferols"[MeSH Terms]) AND ("parkinson disease"[MeSH Terms] OR ("parkinson"[All Fields] AND "disease"[All Fields]) OR "parkinson disease"[All Fields] OR "parkinson s"[All Fields] OR "parkinsons"[All Fields] OR "parkinson"[All Fields] OR "parkinsonian disorders"[MeSH Terms] OR ("parkinsonian"[All Fields] AND "disorders"[All Fields]) OR "parkinsonian disorders"[All Fields] OR "parkinsonism"[All Fields] OR "parkinsonisms"[All Fields] OR "parkinsons s"[All Fields])	438
Scopus	
(TITLE-ABS-KEY("Vitamin D") AND TITLE-ABS-KEY("Parkinson*"))<br>AND (TITLE-ABS-KEY(deficien* OR insufficien* OR sufficien*))<br>AND (TITLE-ABS-KEY("randomized controlled trial" OR "RCT" OR "placebo"))<br>OR (TITLE-ABS-KEY("vitamin D receptor" OR "VDR" OR "polymorphism" OR "genotype"))	137
Google Scholar	
Vitamin D (AND) Parkinson. Use the “Sort by relevance” option (default setting).	2000
Total	2575

https://doi.org/10.3390/neurosci6040130

TABLE S3

Table S3. Chromosome 12 SNPs Associated with the Vitamin D Receptor.

Position (Chr12)	Name	rsID	Alleles	Consequence	Alleles in current study[#]	
					Dominant (A)	Recessive (a)
47906043	A-1012G	rs4516035	T>C	2KB Upstream	T	C
47845054	ApaI	rs7975232	C>A	Intron	A=T	C=G
47844438	BglI	rs739837	G>C / G>T	Prime UTR	T	G
47846052	BsmI	rs1544410	C>A / C>G / C>T	Intron	G=C	A=T
47908762	Cdx2	rs11568820	C>A / C>G / C>T	Promoter region	G	A
47879112	FokI	rs2228570	A>C / A>G / A>T	Initiator Codon	C=G	T=A
47844974	TaqI	rs731236	A>C / A>G / A>T	Missense Variant	T=A	C=G
47845892	Tru9I	rs757343	C>T	Intron	G	A
47884227	NA	rs1989969	A>C / A>G / A>T	Intron	A	G
47889022	NA	rs2853559	A>G	Intron	G	A
47892232	NA	rs4334089	G>A	Intron	C=G	T=A
47902485	NA	rs7299460	C>T	Intron	C	T
47838310	NA	rs7968585	C>G / C>T	Non coding transcript	C	T
47910769	NA	rs7976091	C>T	NA	C=G	T
47902182	NA	rs10083198	T>A / T>C	Intron	C	T

Abbreviations: NA, not applicable/ not available. Adenine (A) pairs with Thymine (T); Cytosine (C) pairs with Guanine (G);

[#]Forward DNA strand

Information obtained from https://www.ncbi.nlm.nih.gov/snp/ (accessed on Aug 26, 2025)

We analyzed the VitD SNPs with snpXplorer by Tesi et al. (2021).

Input: rs4516035, rs7975232, rs739837, rs1544410, rs11568820, rs2228570, rs731236, rs757343, rs1989969, rs2853559, rs4334089, rs7299460, rs7968585, rs7976091, rs10083198.

Enrichment analysis revealed association with vitamin D receptor pathway, vitamin D sensitive calcium signaling, vitamin D metabolism, and vitamin D action mechanisms.

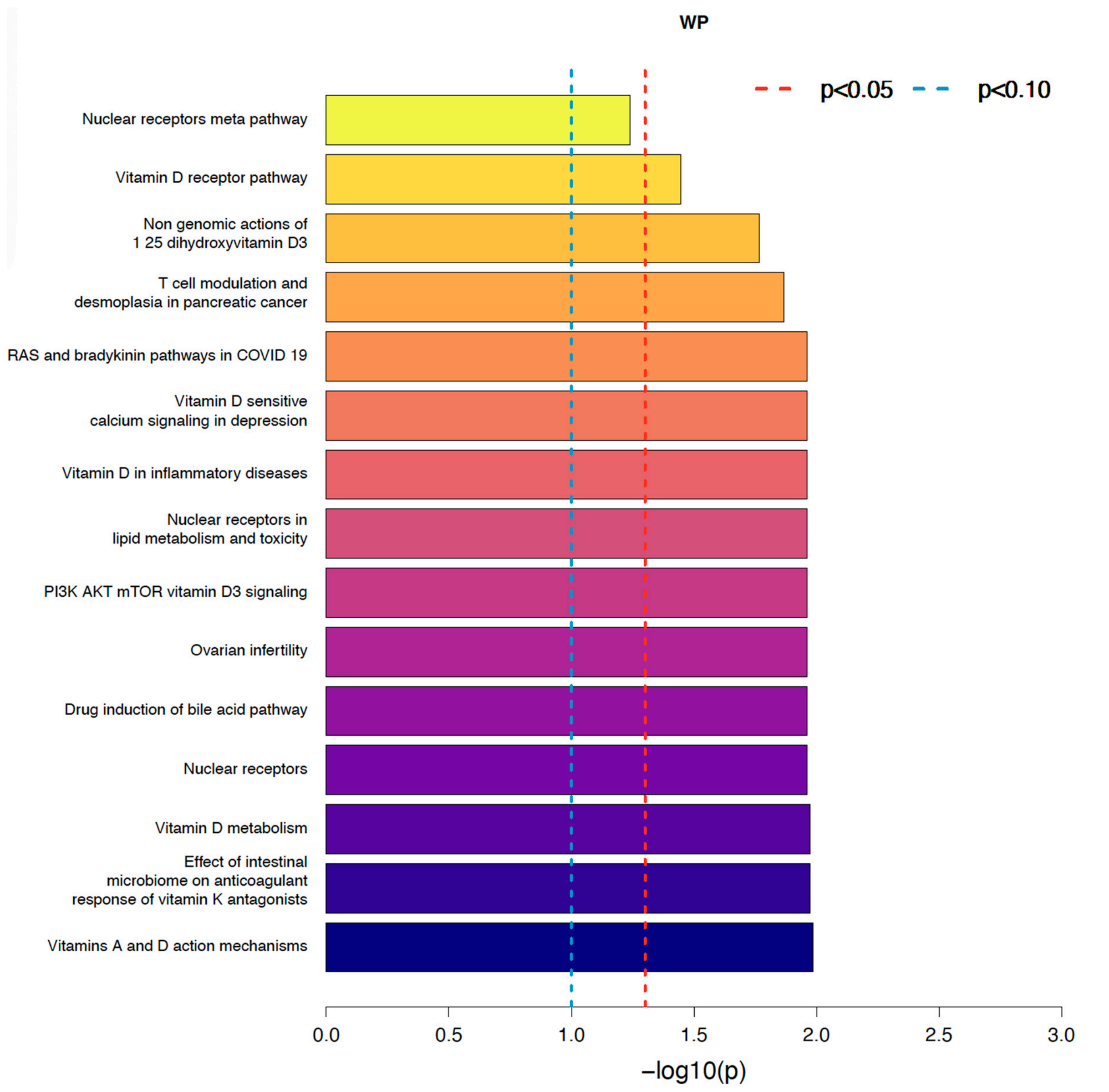

The Gene Ontology (GO) terms derived from the snpXplorer® dataset display distinct functional clusters, indicating enrichment in biologically related processes. The visualization, arranged in both vertical and horizontal orientations, highlights direct associations between individual SNPs and specific GO terms. However, minimal interaction is observed among different GO terms, suggesting that each SNP is likely linked to a discrete genetic function. This pattern implies limited functional overlap, reducing the necessity for investigating SNP triplets or combinations, as the pathways involved appear to operate independently.

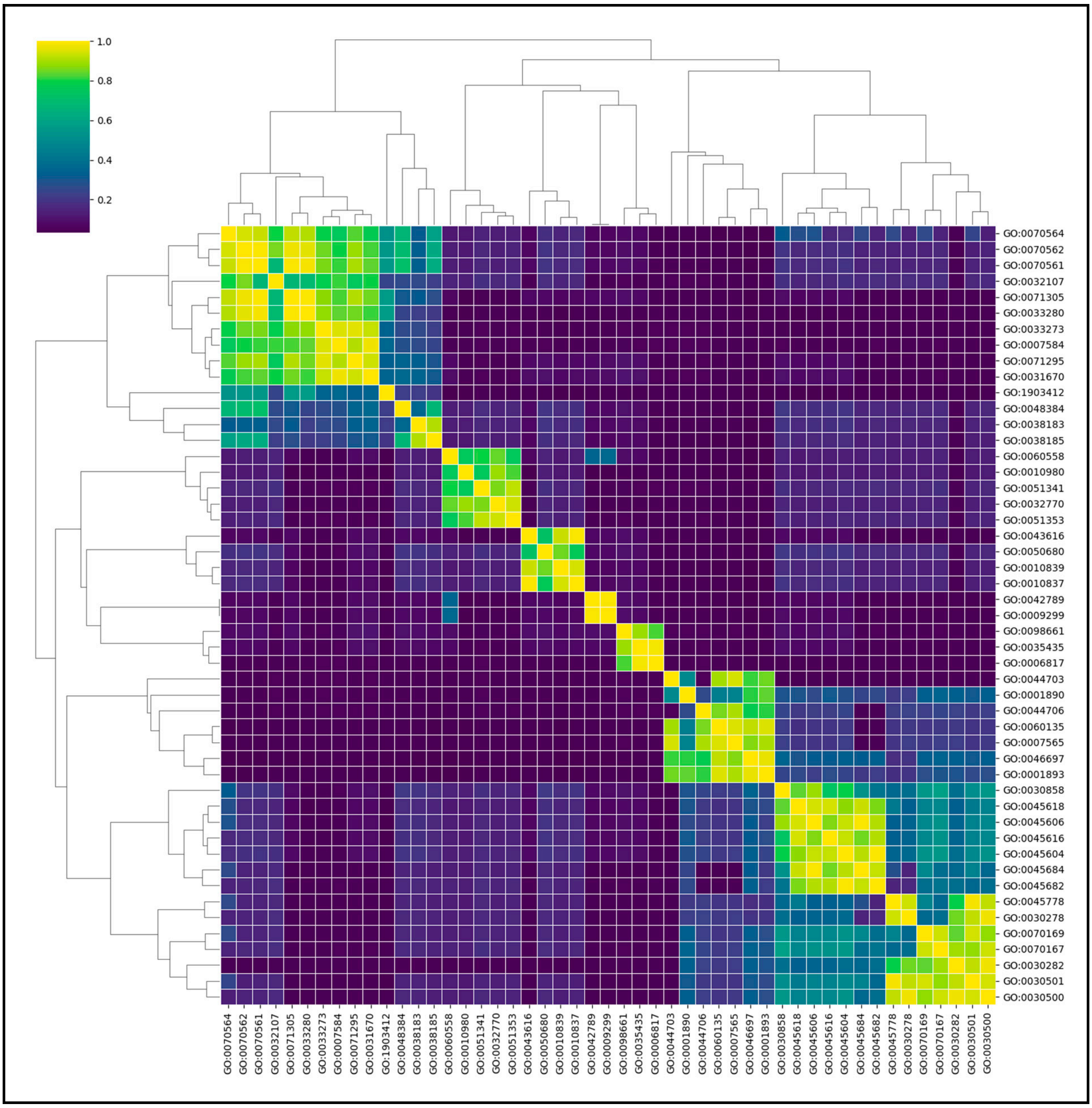

TABLE S4

Table S4. VitD assay methods.

Assay	Principle	Sensitivity & specificity	Advantages & limitations	Reference
CBPA	Competitive binding with labeled ligand	Moderate / Low–Moderate	Simple, low cost; cross-reactivity, outdated	Abou-Raya
ELISA	Antibody-based (enzyme label)	Moderate–High / Moderate–High	Widely available; manual steps, limited dynamic range	Evatt et al. (
ECL	Antibody-based (electrochemiluminescence)	High / High	Wide dynamic range, automated; platform-dependent	van den Bos

CMIA	Antibody-based (chemiluminescent micro-particles)	High / High	High throughput, automation; platform lock-in	Beyazal et a
RIA	Antibody-based (radioisotope label)	Very High / Moderate–High	Very sensitive; radiation hazards, regulatory burden	Yoon et al. (
LC	Chromatographic separation + UV/FLD	Variable / Moderate	Good for chromophoric analytes; lower specificity	Wang et al.
LC-MS	LC + single MS	High / High	Structural specificity; higher cost	Ding et al. (
LC-MS/MS	LC + tandem MS	Very High / Very High	Gold standard for small molecules; expensive, complex	Wang et al.

Abbreviations: CPBA, competitive protein binding assay; ELISA, enzyme-linked immunosorbent assay; ECL, electrochemiluminescence immunoassay; CMIA, chemiluminescent r RIA, radioimmunoassay; LC, liquid chromatography; LC-MS, liquid chromatography–mass spectrometry; LC-MS/MS, liquid chromatography–tandem mass spectrometry.

TABLE S5

Table S5. Quality assessment of observational studies of vitamin D levels based on Newcastle-Ottawa Scale.

Reference	Type of study	Country	Newcastle-Ottawa Scale								
			1	2	3	4	5	6	7	8	Total
Evatt et al. (2008) [2]	Case-control	USA	1	1	1	1	2	1	1	0	8
Abou-Raya et al. (2009) [1]	Case-control	Egypt	1	1	1	1	2	1	1	0	8
Knekt et al. (2010) [9]	Cohort	Finland	1	1	1	1	2	1	1	1	9
Topal et al. (2010) [10]	Case-control	Turkey	1	1	1	1	1	1	0	0	6
Senel et al. (2011) [11]	Case-control	Turkey	1	1	1	1	1	1	0	0	6
Ding et al. (2013) [7]	Cross-sectional	USA	1	1	1	1	2	1	1	0	8
Meamar et al. (2013) [12]	Case-control	Iran	1	1	1	1	2	1	1	0	8
van den Bos et al. (2013) [3]	Cross-sectional	Netherlands	1	1	1	1	2	1	1	0	8
Petersen et al. (2014) [13]	Case-control	Faroe Islands	1	1	1	1	2	1	1	0	8
Liu et al. (2014) [14]	Case-control	China	1	1	1	1	2	1	1	0	8
Wang et al. (2015) [8]	Case-control	USA	1	1	1	1	2	1	1	0	8
Yoon et al. (2015) [5]	Case-control	South Korea	1	1	1	1	2	1	0	0	7
Ozturk et al. (2016) [15]	Case-control	Turkey	1	1	1	1	2	1	0	0	7

Beyazal et al. (2016) [4]	Case-control	Turkey	1	1	1	1	2	1	1	0	8
Wang et al. (2016) [6]	Case-control	China	1	1	1	1	2	1	1	0	8
Hatem et al. (2017) [16]	Case-control	Iraq	1	1	1	1	2	1	0	0	7
Sleeman et al. (2017) [17]	Cohort	UK	1	1	1	1	2	1	1	1	9
Ahangar et al. (2018) [18]	Case-control	Iran	1	1	1	1	2	1	0	0	7
Mollenhauer et al. (2019) [19]	Cohort	Germany	1	1	1	1	2	1	1	1	9
Soliman et al. (2019) [20]	Case-control	Egypt	1	1	1	1	2	1	0	0	7
Zhang et al. (2019) [21]	Case-control	China	1	1	1	1	2	1	1	0	8
Fahmy et al. (2020) [22]	Case-control	Egypt	1	1	1	1	1	1	1	0	7
Ogura et al. (2021) [23]	Case-control	Japan	1	1	1	1	2	1	1	0	8
Barichella et al. (2022) [24]	Cross-sectional	Italy	1	1	1	1	2	1	1	0	8
Kakimoto et al. (2022) [25]	Cross-sectional	Japan	1	0	0	1	0	1	1	0	4
Novotnij et al. (2022) [26]	Case-control	Russia	1	1	1	1	2	1	1	0	8
Wu et al. (2022) [27]	Cross-sectional	China	1	1	0	0	1	1	1	0	5
Yakşi et al. (2022) [28]	Case-control	Turkey	1	1	1	0	1	0	1	1	6
Džoljić et al. (2023) [29]	Case-control	Serbia	1	1	1	1	1	1	1	1	8
Sooragonda et al. (2023) [30]	Case-control	India	1	1	1	1	1	0	1	1	7
Xia et al. (2023) [31]	Case-control	China	1	1	1	1	1	0	1	1	7
Khan et al. (2024) [32]	Case-control	Bangladesh	1	0	1	1	1	0	1	1	6
Milanowski et al. (2024) [33]	Cross-sectional	Poland	1	0	1	1	1	0	1	1	6
Rahman et al. (2024) [34]	Case-control	Bangladesh	1	0	0	1	1	1	1	1	6
Xu et al. (2024) [35]	Case-control	China	1	0	1	1	1	1	1	1	7

1) Representativeness of the Exposed Cohort – Whether the cohort is truly representative of the average population. 2) Selection of the Non-Exposed Cohort – Whether the comparison group is drawn from the same population. 3) Ascertainment of Exposure – How exposure (e.g., vitamin D levels) was determined (e.g., secure records or structured interviews). 4) Demonstration That Outcome Was Not Present at Start – Ensures that the outcome (e.g., PD) was not already present at baseline. 5) Comparability of Cohorts on the Basis of Design or Analysis – Whether the study controlled for confounding factors (e.g., age, sex, lifestyle). This item can receive up to two stars. 6) Assessment of Outcome – How the outcome (e.g., motor symptoms, PD diagnosis) was measured (e.g., independent blind assessment). 7) Was Follow-Up Long Enough for Outcomes to Occur – Whether the follow-

up period was sufficient to observe the outcome. 8) Adequacy of Follow-Up of Cohorts – Whether all or most participants were followed up, and losses were accounted for.

https://doi.org/10.3390/neurosci6040130

TABLE S6

Table S6. Quality assessment of clinical trial studies based on robvis.

Reference	Country	D1	D2	D3	D4	D5	Overall
Dubose et al. (2011) [36]	USA	+	-	-	-	-	Some concerns
Suzuki et al. (2013) [37]	Japan	+	+	+	+	+	Low risk
Habibi et al. (2018) [38]	Iran	+	+	+	-	-	Some concerns
Hiller et al. (2018) [39]	USA	+	-	-	+	-	Low
Barichella et al. (2019) [40]	Italy	+	-	+	+	+	Some concern
Bytowska et al. (2023) [41]	Poland	+	-	x	+	-	High risk
Zali et al. (2024) [42]	Iran	-	+	+	+	-	Some concerns
Li et al. (2025) [43]	China	+	-	+	+	-	Some concerns

Judgment: x, high; -, some concern; +, low. D1, bias arising from the randomization process; D2, Bias due to deviations from intended interventions; D3, Bias due to missing outcome data; D4, Bias in measurement of the outcome; D5, Bias in selection of the reported result

https://doi.org/10.3390/neurosci6040130

TABLE S7

Table S7. Quality assessment of observational studies of genetic polymorphism based on Newcastle-Ottawa Scale.

Reference	Country	NOS								
		1	2	3	4	5	6	7	8	Total
Kim et al. (2005) [44]	South Korea	1	0	1	1	1	0	1	1	6
Han et al. (2012) [45]	China	1	0	1	1	1	0	1	1	6
Liu et al. (2013) [46]	China	1	0	1	1	1	0	1	1	6
Lv et al. (2013) [47]	China	1	0	1	1	1	0	1	1	6
Török et al. (2013) [48]	Hungary	1	0	1	1	1	0	1	1	6
Lin et al. (2014) [49]	Taiwan	1	0	1	1	1	0	1	1	6
Petersen et al. (2014) [13]	Faroe Islands	1	1	1	1	1	1	1	1	8
Gatto et al. (2015) [50]	USA	1	1	1	1	1	1	1	1	8
Fazeli et al. (2016) [51]	Iran	1	0	1	1	1	0	1	1	6
Kang et al. (2016) [52]	South Korea	1	0	1	1	1	1	1	1	7
Meamar et al. (2016) [53]	Iran	1	0	1	1	1	0	1	1	6
Mohammadzadeh et al. (2016) [54]	Iran	1	0	1	1	1	0	1	1	6
Gezen-Ak et al. (2017) [55]	Turkey	1	0	1	1	1	0	1	1	6
Tanaka et al. (2017) [56]	Japan	1	1	1	1	1	1	1	1	8
Hu et al. (2020) [57]	China	1	1	1	1	1	1	1	1	8
Agliardi et al. (2021) [58]	Italy	1	0	1	1	1	0	1	1	6
Fahmy et al. (2021) [59]	Egypt	1	0	1	1	0	0	1	1	5
Redenšek et al. (2022) [60]	Slovenia	1	1	1	1	1	0	1	1	7
Canales-Cortés et al. (2024) [61]	Spain	1	0	1	1	0	0	1	1	5
Kundu et al. (2025) [62]	Bangladesh	1	0	1	1	1	0	1	1	6

1) Representativeness of the Exposed Cohort – Whether the cohort is truly representative of the average population. 2) Selection of the Non-Exposed Cohort – Whether the comparison group is drawn from the same population. 3) Ascertainment of Exposure – How exposure (e.g., vitamin D levels) was determined (e.g., secure records or structured interviews). 4) Demonstration That Outcome Was Not Present at Start –

https://doi.org/10.3390/neurosci6040130

Ensures that the outcome (e.g., PD) was not already present at baseline. 5) Comparability of Cohorts on the Basis of Design or Analysis – Whether the study controlled for confounding factors (e.g., age, sex, lifestyle). This item can receive up to two stars. 6) Assessment of Outcome – How the outcome (e.g., motor symptoms, PD diagnosis) was measured (e.g., independent blind assessment). 7) Was Follow-Up Long Enough for Outcomes to Occur – Whether the follow-up period was sufficient to observe the outcome. 8) Adequacy of Follow-Up of Cohorts – Whether all or most participants were followed up, and losses were accounted for.

TABLE S8

Table S8. Serum 25(OH)D levels in PD versus control

References	Sample Size		25(OH)D levels (ng/mL)		SMD (95% CI)
	PD	HC	PD	HC	
Evatt et al. (2008) [2]	100	99	31.9±13.6	37.0±14.5	-0.36 (-0.64, -0.08)
Abou-Raya et al. (2009) [1]	82	68	12.9±9.9	21.6±4.8	-1.09 (-1.43, -0.74)
Knekt et al. (2010) [9]	50	3123	14.5±7.4	16.7±7.8	-0.28 (-0.56, 0.00)
Topal et al. (2010)[a] [10]	48	50	27.5±7.8	33.8±7.8	-0.80 (-1.21, -0.38)
Senel et al. (2011)[a] [11]	19	20	12.6±9.7	20.4±9.7	-0.80 (-1.4, -0.14)
Ding et al. (2013) [7]	186	802	30.5±12	32.5±10.6	-0.18 (-0.34, -0.02)
van den Bos et al. (2013) [3]	388	283	19.3±8	22.7±9.17	-0.40 (-0.55, -0.24)
Petersen et al. (2014) [13]	80	154	15.7±9.6	15.4±9.9	0.03 (-0.24, 0.30)
Liu et al. (2014) [14]	229	120	20.6±5.5	22.9±5.6	-0.41 (-0.64, -0.19)
Wang et al. (2015) [8]	478	431	25.5±10.9	30.1±10.3	-0.43 (-0.56, -0.30)
Yoon et al. (2015) [5]	81	52	21.8±9.5	25.2±9.3	-0.36 (-0.71, -0.01)
Ozturk et al. (2016) [15]	52	39	12±7.1	17.3±10.6	-0.60 (-1.02, -0.17)
Beyazal et al. (2016) [4]	115	117	13.4±5.2	19.1±6.7	-0.95 (-1.22, -0.67)
Wang et al. (2016) [6]	201	199	20.6±6.5	22.8±5.5	-0.36 (-0.56, -0.17)
Hatem et al. (2017) [16]	40	40	18.0±8.5	24.8±9.1	-0.77 (-1.22, -0.31)
Sleeman et al. (2017) [17]	145	94	17.6±8.6	20.9±8.8	-0.38 (-0.64, -0.12)
Ahangar et al. (2018) [18]	50	50	27.2±27.7	32.0±17.7	-0.20 (-0.60, 0.18)
Mollenhauer et al. (2019) [19]	135	109	18.0±9.1	23.1±8.7	-0.57 (-0.82, -0.31)

https://doi.org/10.3390/neurosci6040130

Soliman et al. (2019) [20]	25	25	23.8±10.3	51.3±22.5	-1.57 (-2.20, -0.93)
Zhang et al. (2019) [21]	182	185	17.3±6.6	19.9±5.6	-0.43 (-0.63, -0.22)
Fahmy et al. (2020) [22]	50	50	6.1±2.5	7.4±3.6	-0.42 (-0.82, -0.02)
Ogura et al. (2021) [23]	27	61	13.3±4.7	26.8±7.6	-1.97 (-2.50, -1.43)
Barichella et al. (2022) [24]	500	100	17.4±9.0	20.2±10.4	-0.30 (-0.51, -0.08)
Kakimoto et al. (2022) [25]	20	19	14.7±4.6	24.3±6.7	-1.66 (-2.39, -0.93)
Novotnij et al. (2022) [26]	138	79	21.3±9.4	24.8±9.5	-0.37 (-0.65, -0.10)
Wu et al. (2022) [27]	112	70	18.3±5.9	22.6±5.6	-0.74 (-1.05, -0.43)
Yakşi et al. (2022) [28]	34	31	16.0±5.1	17.7±9.2	-0.23 (-0.72, 0.25)
Džoljić et al. (2023) [29]	113	82	23.6±3.3	23.5±2.1	0.03 (-0.24, 0.31)
Sooragonda et al. (2023) [30]	40	40	22.1±9.2	34.1±11.8	-1.13 (-1.60, -0.66)
Xia et al. (2023) [31]	100	100	32.3±13.9	42.54±17.7	-0.63 (-0.92, -0.35)
Milanowski et al. (2024) [33]	26	26	22.9±5.6	27.9±9.2	-0.65 (-1.21, -0.09)
Rahman et al. (2024) [34]	50	50	18.5±5.9	29.4±5.9	-1.84 (-2.31, -1.37)
Xu et al. (2024) [35]	24	24	16.7±6.4	20.2±2.3	-0.72 (-1.30, -0.13)
[a]estimated SD based on large effect size. Note: Khan et al. (2024) was removed from the table due to significant outlier results [32].					

https://doi.org/10.3390/neurosci6040130

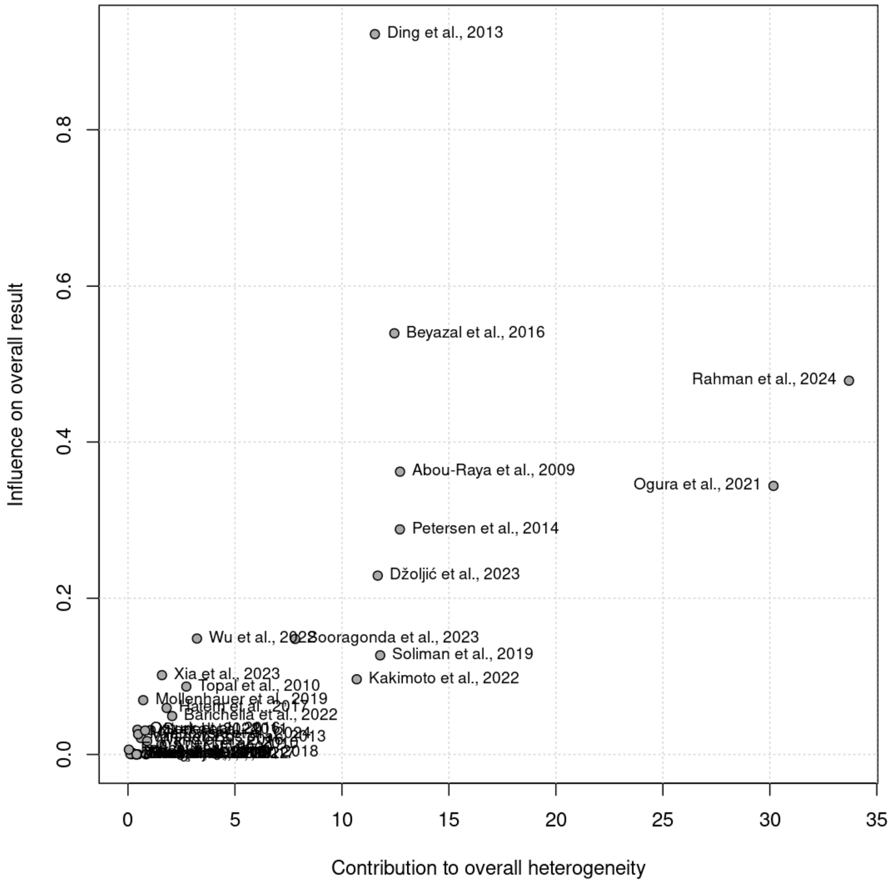

Figure. Baujat Plot of VitD levels in patients with PD versus controls. Identifies studies that contribute to heterogeneity

TABLE S9

Table S9. 25(OH)D insufficiency in PD versus control					
References	Sample Size		25(OH) insufficiency		OR (95% CI)
	PD	HC	PD	HC	
Evatt et al. (2008) [2]	100	99	55	36	2.14 (1.21, 3.78)
Ding et al. (2013) [7]	388	283	182	112	1.34 (0.98, 1.83)
van den Bos et al. (2013) [3]	186	802	104	346	1.67 (1.21, 2.31)
Wang et al. (2015) [8]	478	431	340	233	2.09 (1.59, 2.75)
Ozturk et al. (2016) [15]	115	117	115	98	5.38 (1.77, 16.36)
Beyazal et al. (2016) [4]	52	39	25	13	1.85 (0.78, 4.38)
Wang et al. (2016) [6]	201	199	183	179	1.14 (0.58, 2.22)
Sleeman et al. (2017) [17]	145	94	132	75	2.57 (1.20, 5.50)
Zhang et al. (2019) [21]	182	185	39	67	0.48 (0.30, 0.76)
Fahmy et al. (2020) [22]	50	50	5	9	0.51 (0.16, 1.63)
Novotnij et al. (2022) [26]	138	79	51	26	1.19 (0.66, 2.14)
Xia et al. (2023) [31]	100	100	27	20	1.47 (0.76, 2.86)
Note: Khan et al. (2024) was removed from the table due to significant outlier results [32].					

https://doi.org/10.3390/neurosci6040130

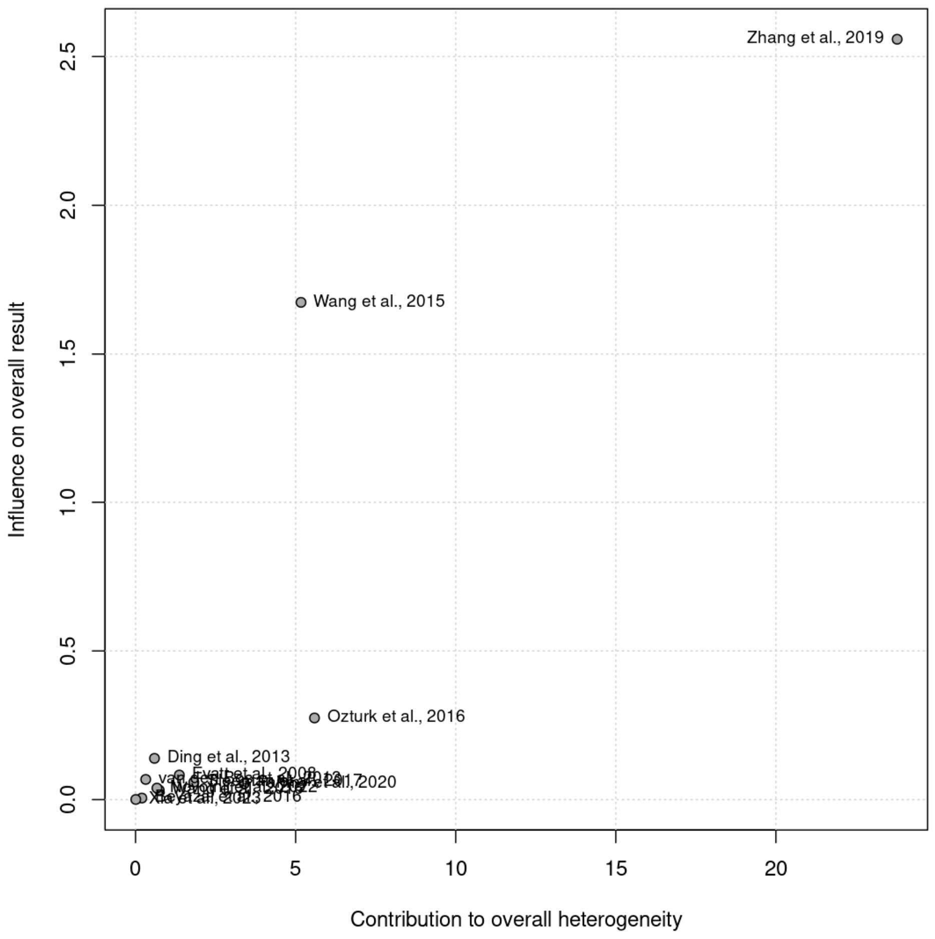

Figure. Baujat Plot of VitD insufficiency in patients with PD versus controls. Identifies studies that contribute to heterogeneity.

TABLE S10

Table S10. 25(OH)D deficiency in PD versus control					
References	Sample Size		25(OH) deficiency		OR (95% CI)
	PD	HC	PD	HC	
Evatt et al. (2008) [2]	100	99	23	10	2.66 (1.19, 5.93)
Ding et al. (2013) [7]	388	283	68	26	2.10 (1.30, 3.40)
Meamar et al. (2013) [12]	85	112	39	68	0.55 (0.31, 0.97)
Liu et al. (2014) [14]	229	120	99	32	2.09 (1.29, 3.39)
Wang et al. (2015) [8]	478	431	172	73	2.76 (2.01, 3.77)
Yoon et al. (2015) [5]	81	52	45	19	2.17 (1.06, 4.44)
Beyazal et al. (2016) [4]	52	39	23	5	5.39 (1.82, 15.99)
Ozturk et al. (2016) [15]	115	117	105	79	5.05 (2.37, 10.75)
Hatem et al. (2017) [16]	40	40	25	11	4.39 (1.70, 11.29)
Sleeman et al. (2017) [17]	145	94	97	43	2.41 (1.41, 4.09)
Soliman et al. (2019) [20]	25	25	21	6	13.12 (3.14, 54.74)
Zhang et al. (2019) [21]	182	185	125	100	1.86 (1.22, 2.85)
Fahmy et al. (2020) [22]	50	50	45	40	2.25 (0.71, 7.14)
Barichella et al. (2022) [24]	500	100	328	53	1.69 (1.09, 2.61)
Novotnij et al. (2022) [26]	138	79	49	24	1.26 (0.69, 2.28)
Xia et al. (2023) [31]	100	100	21	4	6.38 (2.10, 19.36)
Khan et al. (2024) [32]	60	60	3	1	3.11 (0.31, 30.74)

https://doi.org/10.3390/neurosci6040130

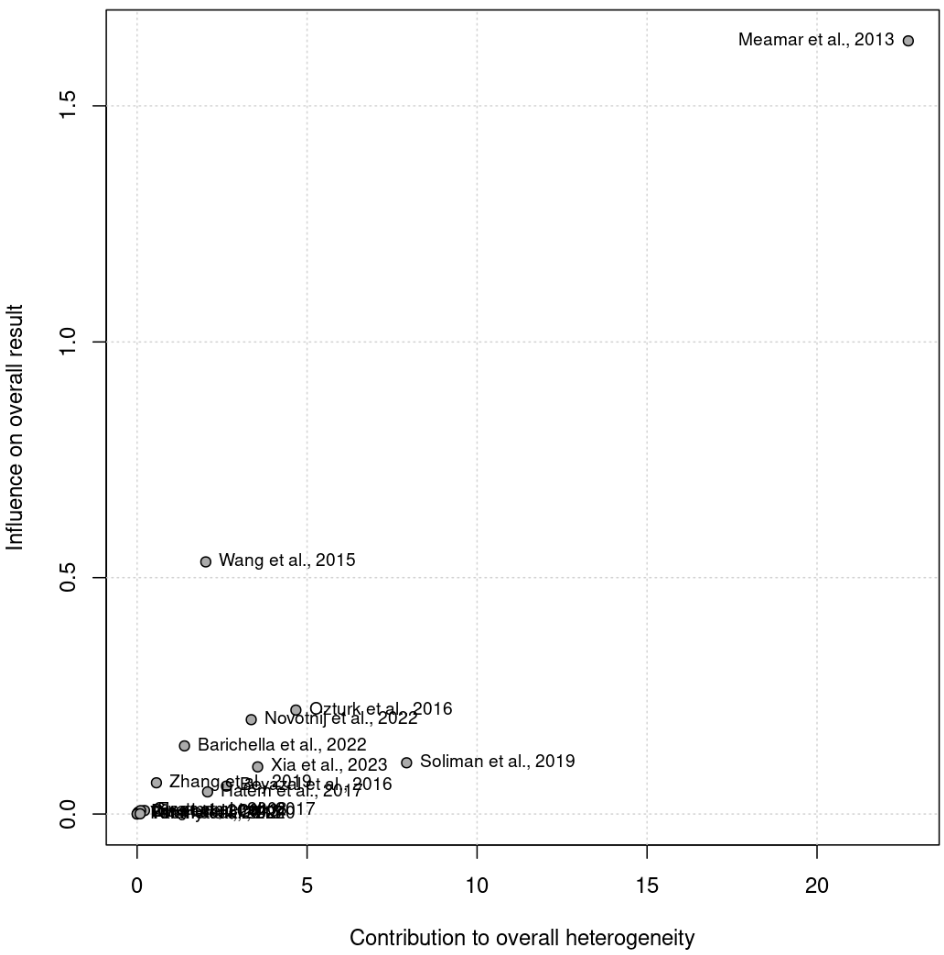

Figure. Baujat Plot of VitD deficiency in patients with PD versus controls. Identifies studies that contribute to heterogeneity.

TABLE S11

Table S11. VitD and PD incidence.

References	Sample Size	PD incident cases	VITD status	HR (95% CI)
Gröninger et al. (2024) [63]	183225	602	Deficient	1.08 (0.80, 1.45)
Veronese et al. (2024) [64]	502027	3325	Sufficient	0.83 (0.74, 0.93)

https://doi.org/10.3390/neurosci6040130

TABLE S12

Table S12. VitD supplementation versus placebo in PD.

References	Sample Size		UPDRS- Total "ON"(Δ)			UPDRS-III during "ON" (Δ)			TUG "ON"(Δ)	
	VitD	PLC	VitD	PLC	SMD (95% CI)	VitD	PLC	SMD (95% CI)	VitD	PLC
Dubose et al. (2011) [36]	16	14	NA	NA	NA	-4.90 ± 13.87	-4.40 ± 16.35	0.03 (-0.68, 0.75)	-0.20 ± 3.26	0.20 ± 5.9[illegible]
Suzuki et al. (2013) [37]	55	57	-0.87 ± 12.8	4.20 ± 14.5	-0.37 (-0.74, 0.003)	-1.05 ± 10.0	1.05 ± 9.09	-0.22 (-0.59, 0.15)	NA	NA
Habibi et al. (2018) [38]	60	60	NA	NA	NA	-1.32 ± 10.47	-2.98 ± 10.11	0.16 (-0.19, 0.52)	NA	NA
Hiller et al. (2018) [39]	27	22	NA	NA	NA	0.48 ± 5.59	-0.18 ± 9.73	0.08 (-0.47, 0.64)	0.09 ± 0.54	-0.06 ± 0.[illegible]
Barichella et al. (2019) [40]	75	75	-14.4 ± 0.66	-14.8 ± 0.89	0.51 (0.18, 0.83)	-7.6 ± 0.51	-7.6 ± 0.61	0.00 (-0.32, 0.32)	-3.8 ± 4.42	-2.65 ± 2.[illegible]
Zali et al. (2024) [42]	23	23	-16.35 ± 26.36	-4.56 ± 10.59	-0.59 (-1.18, 0.00)	-2.82 ± 12.18	0.35 ± 4.25	-0.35 (-0.93, 0.23)	NA	NA
Bytowska et al. (2023) [41]	13	16	NA	NA	NA	NA	NA	NA	-2.23 ± 6.36	-0.79 ± 2.[illegible]
Li et al. (2025) [43]	15	15	-4.73 ± 5.19	0.73 ± 2.52	-1.34 (-2.13, -0.55)	-4.27 ± 3.77	0.40 ± 1.84	-1.57 (-2.39, -0.76)	NA	NA

https://doi.org/10.3390/neurosci6040130

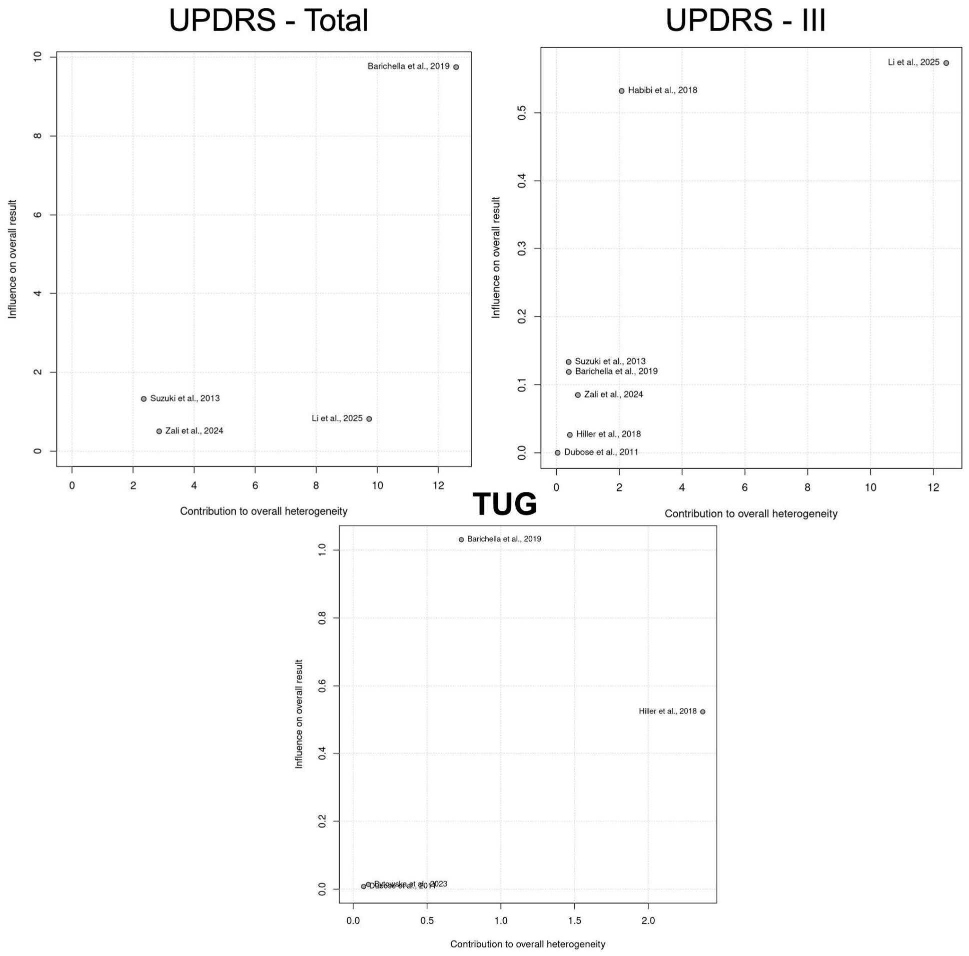

Figure. Baujat plot of the effect of VitD on motor symptoms in patients with PD compared to healthy controls. Identifies studies that contribute to heterogeneity.

TABLE S13

Table S13. Allele and genotype frequency of HC and PD patients.

Reference	Sample size	Allele frequency					Genotype frequency			
	Case/ Control	Case		Control		HWE[i]	Case			Control
		■	○	■	○		■■	■○	○○	■■
**ApaI (rs7975232) (■=A=T; ○=C=G)**										
Liu et al. (2013)[iii] [46]	285/285	NA	NA	NA	NA	NA	130	135	20	149
Petersen et al. (2014)[iv] [13]	121/235	130	112	236	232	0.42	34	62	25	58
Gatto et al. (2015)[v] [50]	282/419	250	314	418	420	**0.04***	46	158	78	104
Meamar et al. (2016) [53]	59/53	58	60	68	38	**0.03***	14	32	13	2
Gezen-Ak et al. (2017) [55]	381/241	454	308	317	165	**0.03***	130	194	57	101
Tanaka et al. (2017)[v] [56]	229/357	NA	NA	NA	NA	NA	18	102	109	32
Agliardi et al. (2021) [58]	406/800	419	393	882	718	0.11	118	183	105	244
Fahmy et al. (2021) [59]	50/50	56	44	63	37	0.38	14	28	8	21
Kundu et al. (2025)[v] [62]	100/100	118	82	94	106	**0.02***	33	52	15	22
Overall	1913/2540	1485	1313	2078	1716	0.17	537	946	430	733
**A-1012G (rs4516035) (■=T; ○=C)**										
Redenšek et al. (2022) [60]	231/161	NA	NA	NA	NA	NA	63	117	51	40
**BglI (rs739837) (■=T; ○=G)**										
Redenšek et al. (2022) [60]	231/161	NA	NA	NA	NA	NA	66	112	53	43
**BsmI (rs1544410) (■=G=C; ○=A=T)**										
Kim et al. (2005) [44]	85/231	15	155	66	396	0.08	2	11	72	3
Han et al. (2012) [45]	260/282	478	42	524	40	0.56	222	34	4	244
Török et al. (2013) [48]	100/109	103	97	111	107	0.92	27	49	24	27

https://doi.org/10.3390/neurosci6040130

Petersen et al. (2014)[iv] [13]	121/235	149	93	285	185	0.87	48	53	20	84
Gatto et al. (2015) [50]	276/416	233	315	319	517	0.11	79	161	36	151
Kang et al. (2016) [52]	137/163	NA	NA	NA	NA	NA	123	13	1	145
Meamar et al. (2016) [53]	59/53	75	43	62	44	0.49	8	27	24	8
Gezen-Ak et al. (2017)[vi] [55]	380/239	406	354	266	212	0.44	136	134	110	94
Tanaka et al. (2017)[v] [56]	229/357	NA	NA	NA	NA	NA	178	45	6	291
Agliardi et al. (2021)[iv] [58]	406/800	429	383	859	741	0.69	131	167	108	276
Redenšek et al. (2022) [60]	231/161	NA	NA	NA	NA	NA	78	119	34	58
Canales-Cortés et al. (2024) [61]	54/16	52	56	25	7	0.08	13	26	15	10
Kundu et al. (2025) [62]	100/100	99	101	81	119	0.08	26	47	27	18
Overall	2438/3165	2039	1639	2598	2368	**< 0.01***	1071	886	481	1409
**Cdx2 (rs11568820) (■=G; ○=A)**										
Gatto et al. (2015) [50]	282/423	453	111	667	179	0.54	183	87	12	266
Redenšek et al. (2022) [60]	231/161	NA	NA	NA	NA	NA	159	64	8	108
Overall	513/584	453	111	667	179	0.54	342	151	20	374
**FokI (rs2228570 aka rs10735810) (■=C=G; ○=T=A)**										
Han et al. (2012) [45]	260/282	352	168	344	220	**0.02***	114	124	22	109
Török et al. (2013) [48]	100/109	132	68	119	99	**0.02***	42	48	10	35
Gatto et al. (2015) [50]	283/422	344	222	509	335	0.86	109	126	48	153
Kang et al. (2016) [52]	137/163	NA	NA	NA	NA	NA	46	63	28	48
Meamar et al. (2016) [53]	59/53	84	34	91	15	**< 0.01***	6	22	31	2
Mohammadzadeh et al. (2016) [54]	150/160	123	27	134	26	0.76	123	27	0	134
Gezen-Ak et al. (2017) [55]	382/237	526	238	317	157	0.49	181	164	37	105
Tanaka et al. (2017)[vi] [56]	229/357	NA	NA	NA	NA	NA	108	98	23	141

https://doi.org/10.3390/neurosci6040130

Hu et al. (2020) [57]	470/470	482	458	541	399	**< 0.01***	131	220	119	149
Agliardi et al. (2021) [58]	406/800	468	344	1067	533	**< 0.01***	136	196	74	362
Fahmy et al. (2021) [59]	50/50	77	23	73	27	0.62	28	21	1	25
Redenšek et al. (2022) [60]	231/161	NA	NA	NA	NA	NA	88	102	41	60
Canales-Cortés et al. (2024) [61]	54/16	75	33	23	9	1.00	27	21	6	8
Kundu et al. (2025) [62]	100/100	52	148	72	128	0.04	11	30	59	8
Overall	2911/3380	2715	1763	3290	1948	**0.02***	1150	1262	499	1339
**TaqI (rs731236) (■=T=A; ○=C=G)**										
Liu et al. (2013)[v] [46]	285/285	NA	NA	NA	NA	NA	252	33	0	255
Lv et al. (2013) [47]	483/498	920	46	944	52	0.67	437	46	0	446
Török et al. (2013) [48]	100/109	118	82	140	78	0.31	35	48	17	47
Petersen et al. (2014)[iv] [13]	121/235	148	94	281	187	0.80	47	54	20	81
Gatto et al. (2015) [50]	282/421	316	248	519	323	**0.04***	77	162	43	153
Kang et al. (2016) [52]	137/163	NA	NA	NA	NA	NA	119	18	0	147
Meamar et al. (2016) [53]	59/53	81	37	72	34	1.00	6	25	28	4
Gezen-Ak et al. (2017) [55]	381/240	490	272	316	164	0.62	154	182	45	109
Tanaka et al. (2017)[v] [56]	229/357	NA	NA	NA	NA	NA	178	47	4	284
Agliardi et al. (2021)[v] [58]	406/800	476	336	961	639	0.51	134	208	64	288
Redenšek et al. (2022) [60]	231/161	NA	NA	NA	NA	NA	84	113	34	60
Canales-Cortés et al. (2024) [61]	54/16	73	35	25	7	0.28	24	25	5	10
Kundu et al. (2025) [62]	100/100	136	64	151	49	0.12	40	56	4	57
Overall	2868/3438	2758	1214	3409	1533	0.64	1587	1017	264	1941
**Tru9I (rs757343) (■=G; ○=A)**										

Gezen-Ak et al. (2017) [55]	381/229	609	153	351	107	0.19	245	119	17	136
**rs1989969 (■=A; ○=G)**										
Agliardi et al. (2021) [58]	406/800	298	514	600	1000	0.72	51	196	159	125
**rs2853559 (■=G; ○=A)**										
Lin et al. (2014) [49]	700/792	NA	NA	NA	NA	NA	305	312	83	314
**rs4334089 (■=C=G; ○=T=A)**										
Lv et al. (2013)[v] [47]	483/498	543	423	556	440	0.89	147	249	87	156
Lin et al. (2014) [49]	700/792	NA	NA	NA	NA	NA	223	361	116	245
Gatto et al. (2015) [50]	283/423	419	147	624	222	0.95	152	115	16	228
Fazeli et al. (2016) [51]	520/1040	471	569	529	511	**0.01***	115	241	165	132
Overall	1986/2753	1433	1139	1709	1173	**< 0.01***	637	966	384	761
**rs7299460 (■=C; ○=T)**										
Lin et al. (2014) [49]	700/792	NA	NA	NA	NA	NA	112	349	239	124
**rs7968585 (■=C; ○=T)**										
Lin et al. (2014) [49]	700/792	NA	NA	NA	NA	NA	369	270	61	421
**rs7976091 (■=C=G; ○=T=A)[vii]**										
Török et al. (2013)[v] [48]	100/109	73	127	88	130	0.42	15	43	42	21
Lin et al. (2014) [49]	700/792	NA	NA	NA	NA	NA	234	361	105	250
Kang et al. (2016)[vii] [52]	137/163	NA	NA	NA	NA	NA	37	78	22	60
Mohammadzadeh et al. (2016) [54]	150/160	114	36	32	128	**< 0.01***	30	84	36	6
Canales-Cortés et al. (2024)[v] [61]	54/16	49	59	18	14	0.31	10	29	15	4
Overall	1141/1240	236	222	138	272	**<0.01***	326	595	220	341
**rs10083198 (■=C; ○=T)**										
Lin et al. (2014) [49]	700/792	NA	NA	NA	NA	NA	213	343	144	259

https://doi.org/10.3390/neurosci6040130

Abbreviations: HWE, Hardy–Weinberg equilibrium of control individuals; NA, no available/ not reported/ not applicable; *, significant results $p < 0.05$; ■, dominant allele (e.g., A

Notes:

i) Fisher's Exact Test.

ii) Pearson chi-square test, df=1.

iii) Data obtained from Geng et al. (2018).

iv) Petersen et al. (2014) incorrectly positioned the percentage symbols (%) and sample sizes (n) in their Table 4 [13].

v) Forward strand was performed, so T allele counts = A allele counts, and G allele counts = C allele counts

vi) A corrected publication has clarified the allele assignments, as the original article appears to have mistakenly reversed the major and minor alleles. HWE was estimated.

vii, rs7976091 is sometimes labeled as ApaI, but this is uncommon; we report it separately from rs7975232

Table. Comparison between alleles and genotypes of VitD SNPs and Parkinson's disease				
Reference	Comparison OR (95% CI and p-value)[i]			
	Allele	Dominant	Recessive	Additive
**ApaI (rs7975232)**				
Liu et al. (2013)[ii] [46]	0.88 (0.68, 1.13), p = 0.36	0.76 (0.55, 1.06), p = 0.11	0.82 (0.44, 1.52), p = 0.62	1.14 (0.88, 1.48), p = 0.31
Petersen et al. (2014) [13]	1.14 (0.83, 1.55), p = 0.40	1.18 (0.72, 1.94), p = 0.49	0.82 (0.48, 1.41), p = 0.48	0.87 (0.63, 1.19), p = 0.39
Gatto et al. (2015) [50]	**0.80 (0.64, 0.99), p = 0.04***	**0.59 (0.40, 0.86), p < 0.01***	1.14 (0.81, 1.60), p = 0.44	**1.26 (1.015, 1.58), p = 0.03***
Meamar et al. (2016) [53]	**0.54 (0.31, 0.92), p = 0.02***	**7.93 (1.70, 36.81), p < 0.01***	0.59 (0.25, 1.39), p = 0.23	**0.44 (0.23, 0.85), p = 0.01***
Gezen-Ak et al. (2017) [55]	**0.76 (0.60, 0.97), p = 0.02***	0.71 (0.51, 1.01), p = 0.05	1.44 (0.86, 2.38), p = 0.15	**1.32 (1.03, 1.69), p = 0.02***
Tanaka et al. (2017)[ii] [56]	0.96 (0.75, 1.24), p = 0.80	0.86 (0.47, 1.58), p = 0.64	1.01 (0.72, 1.40), p = 0.95	1.03 (0.79, 1.34), p = 0.80
Agliardi et al. (2021) [58]	0.86 (0.73, 1.02), p = 0.10	0.93 (0.71, 1.21), p = 0.60	**1.37 (1.03, 1.82), p = 0.02***	1.14 (0.97, 1.35), p = 0.10
Fahmy et al. (2021) [59]	074 (0.42, 1.31), p = 0.31	0.53 (0.23, 1.23), p = 0.14	1.00 (0.34, 2.91), p = 1.00	1.34 (0.75, 2.39), p = 0.31
Kundu et al. (2025) [62]	**1.62 (1.09, 2.41), p = 0.01***	1.74 (0.92, 3.28), p = 0.08	**0.45 (0.22, 0.91), p = 0.02***	**0.60 (0.40, 0.91), p = 0.01***
Overall	0.93 (0.84, 1.03), p = 0.17	0.96 (0.84, 1.09), p = 0.55	0.94 (0.82, 1.09), p = 0.47	0.99 (0.91, 1.08), p = 0.95
**A-1012G (rs4516035)**				
Redenšek et al. (2022)[ii] [60]	1.00 (0.75, 1.33), p = 0.97	1.13 (0.71, 1.79), p = 0.59	1.14 (0.69, 1.87), p = 0.59	0.99 (0.74, 1.33), p = 0.97
**BglI (rs739837)**				
Redenšek et al. (2022)[ii] [60]	1.06 (0.80, 1.41), p = 0.66	1.09 (0.69, 1.72), p = 0.68	0.93 (0.58, 1.49), p = 0.76	0.94 (0.70, 1.24), p = 0.66
**BsmI (rs1544410)**				
Kim et al. (2005) [44]	0.58 (032, 1.04), p = 0.07	1.83 (0.30, 11.15), p = 0.51	**2.07 (1.07, 4.00), p = 0.02***	1.73 (0.95, 3.13), p = 0.07
Han et al. (2012) [45]	0.86 (0.55, 1.36), p = 0.54	0.90 (0.56, 1.47), p = 0.70	2.18 (0.39, 12.04), p = 0.36	1.13 (0.73, 1.75), p = 0.55
Török et al. (2013) [48]	1.02 (0.69, 1.50), p = 0.11	1.12 (0.60, 2.08), p = 0.71	1.06 (0.55, 2.01), p = 0.85	0.97 (0.66, 1.43), p = 0.90
Petersen et al. (2014) [13]	1.04 (0.75, 1.42), p = 0.80	1.18 (0.75, 1.85), p = 0.46	1.17 (0.64, 2.16), p = 0.60	0.96 (0.69, 1.32), p = 0.80

https://doi.org/10.3390/neurosci6040130

Gatto et al. (2015) [50]	1.19 (0.96, 1.49), p = 0.10	**0.70 (0.50, 0.97), p = 0.03***	1.09 (0.69, 1.73), p = 0.68	1.23 (0.97, 1.56), p = 0.08
Kang et al. (2016)[ii] [52]	1.06 (0.55, 2.14), p = 1.00	1.09 (0.52, 2.28), p = 0.81	1.19 (0.07, 19.22), p = 0.90	0.93 (0.47, 1.84), p = 0.85
Meamar et al. (2016) [53]	1.23 (0.72, 2.12), p = 0.43	0.88 (0.30, 2.54), p = 0.81	1.45 (0.66, 3.15), p = 0.34	1.24 (0.71, 2.16), p = 0.43
Gezen-Ak et al. (2017) [55]	0.91 (0.72, 1.15), p = 0.44	0.85 (0.61, 1.20), p = 0.37	1.04 (0.73, 1.49), p = 0.80	1.07 (0.87, 1.30), p = 0.50
Tanaka et al. (2017)[ii] [56]	0.78 (0.54, 1.14), p = 0.21	0.79 (0.52, 1.19), p = 0.26	1.57 (0.50, 4.94), p = 0.43	1.24 (0.87, 1.77), p = 0.22
Agliardi et al. (2021) [58]	0.96 (0.81, 1.14), p = 0.69	0.90 (0.70, 1.16), p = 0.43	0.97 (0.74, 1.27), p = 0.84	1.02 (0.88, 1.19), p = 0.71
Redenšek et al. (2022)[ii] [60]	1.03 (0.77, 1.37), p = 0.88	0.89 (0.58, 1.36), p = 0.61	0.74 (0.43, 1.28), p = 0.29	0.96 (0.72, 1.29), p = 0.82
Canales-Cortés et al. (2024) [61]	**0.26 (0.10, 0.65), p < 0.01***	**0.19 (0.05, 0.62), p < 0.01***	5.76 (0.69, 47.58), p = 0.10	**3.64 (1.41, 9.38), p < 0.01***
Kundu et al. (2025) [62]	1.44 (0.96, 2.13), p = 0.07	1.60 (0.81, 3.15), p = 0.17	0.62 (0.34, 1.14), p = 0.13	0.70 (0.48, 1.04), p = 0.08
Overall	**1.13 (1.04, 1.23), p < 0.01***	0.97 (0.87, 1.08), p = 0.63	0.94 (0.82, 1.07), p = 0.36	0.99 (0.92, 1.06), p = 0.86
**Cdx2 (rs11568820)**				
Gatto et al. (2015) [50]	1.09 (0.84, 1.42), p = 0.50	1.09 (0.79, 1.49), p = 0.58	0.81 (0.39, 1.66), p = 0.56	0.91 (0.70, 1.18), p = 0.50
Redenšek et al. (2022)[ii] [60]	0.98 (0.67, 1.43), p = 1.00	1.08 (0.70, 1.66), p = 0.71	2.85 (0.59, 13.61), p = 0.18	1.01 (0.69, 1.49), p = 0.93
Overall	1.09 (0.84, 1.42), p = 0.50	1.12 (0.87, 1.44), p = 0.36	0.94 (0.51, 1.73), p = 0.85	0.91 (0.74, 1.12), p = 0.40
**FokI (rs2228570 aka rs10735810)**				
Han et al. (2012) [45]	**1.34 (1.04, 1.72), p = 0.02***	1.23 (0.87, 1.74), p = 0.21	**0.46 (0.27, 0.79), p < 0.01***	**0.74 (0.57, 0.95), p = 0.02***
Török et al. (2013) [48]	**1.61 (1.07, 2.39), p = 0.01***	1.53 (0.87, 2.69), p = 0.13	**0.37 (0.16, 0.82), p = 0.01***	**0.62 (0.42, 0.92), p = 0.02***
Gatto et al. (2015) [50]	1.01 (0.82, 1.26), p = 0.85	1.10 (0.80, 1.50), p = 0.54	1.10 (0.73, 1.65), p = 0.64	0.98 (0.79, 1.21), p = 0.86
Kang et al. (2016)[ii] [52]	1.12 (0.81, 1.55), p = 0.51	1.21 (0.74, 1.97), p = 0.44	0.90 (0.51, 1.58), p = 0.72	0.89 (0.65, 1.22), p = 0.48
Meamar et al. (2016) [53]	**0.40 (0.20, 0.80), p < 0.01***	2.88 (0.55, 14.96), p = 0.20	**0.35 (0.16, 0.80), p = 0.01***	**0.44 (0.22, 0.85), p = 0.01***
Mohammadzadeh et al. (2016) [54]	0.88 (0.48, 1.59), p = 0.68	0.88 (0.48, 1.59), p = 0.68	1.06 (0.02, 54.08), p = 0.97	1.13 (0.62, 2.04), p = 0.68
Gezen-Ak et al. (2017) [55]	1.09 (0.85, 1.39), p = 0.46	1.13 (0.81, 1.56), p = 0.45	0.90 (0.53, 1.55), p = 0.72	0.91 (0.71, 1.16), p = 0.46
Tanaka et al. (2017)[ii] [56]	1.27 (0.99, 1.63), p = 0.05	1.34 (0.96, 1.87), p = 0.08	0.73 (0.43, 1.24), p = 0.25	0.78 (0.61, 1.00), p = 0.05

Hu et al. (2020) [57]	**0.77 (0.64, 0.93), p < 0.01***	0.83 (0.62, 1.10), p = 0.19	**1.70 (1.23, 2.34), p < 0.01***	**1.28 (1.07, 1.54), p < 0.01***
Agliardi et al. (2021) [58]	**0.67 (0.57, 0.80), p < 0.01***	**0.60 (0.47, 0.78), p < 0.01***	**1.65 (1.18, 2.30), p < 0.01***	**1.45 (1.22, 1.73), p < 0.01***
Fahmy et al. (2021) [59]	1.23 (0.65, 2.35), p = 0.51	1.27 (0.57, 2.79), p = 0.54	3.06 (0.12, 76.95), p = 0.49	0.86 (0.40, 1.83), p = 0.70
Redenšek et al. (2022)[ii] [60]	0.87 (0.65, 1.17), p = 0.37	1.03 (0.68, 1.56), p = 0.86	1.82 (0.99, 3.34), p =0.05	1.14 (0.85, 1.53), p = 0.36
Canales-Cortés et al. (2024) [61]	0.88 (0.37, 2.12), p = 0.79	1.00 (0.32, 3.05), p = 1.00	1.87 (0.20, 16.83), p = 0.57	1.11 (0.47, 2.62), p = 0.79
Kundu et al. (2025) [62]	**0.62 (0.40, 0.95), p = 0.03***	1.42 (0.54, 3.69), p = 0.47	**2.55 (1.44, 4.52), p < 0.01***	**1.61 (1.04, 2.49), p = 0.03***
Overall	**0.91 (0.84, 0.98), p = 0.02***	0.99 (0.89, 1.10), p = 0.92	**1.15 (1.01, 1.32), p = 0.03***	1.04 (0.97, 1.11), p = 0.24
**TaqI (rs731236)**				
Liu et al. (2013)[ii] [46]	0.90 (0.54, 1.50), p = 0.79	0.89 (0.53, 1.51), p = 0.40	1.00 (0.01), 50.57), p = 1.00	1.11 (0.65, 1.88), p = 0.068
Lv et al. (2013) [47]	1.10 (0.73, 1.65), p = 0.64	1.10 (0.72, 1.68), p = 0.63	1.03 (0.02, 52.06), p = 0.98	0.90 (0.59, 1.37), p = 0.63
Török et al. (2013) [48]	0.80 (0.54, 1.19), p = 0.27	0.71 (0.40, 1.24), p = 0.23	1.19 (0.56, 2.50), p = 0.64	1.23 (0.83, 1.81), p = 0.28
Petersen et al. (2014) [13]	1.04 (0.76, 1.43), p = 0.77	1.19 (0, 76, 1.88), p = 0.43	1.16 (0.63, 2.12), p = 0.61	0.95 (0.69, 1.31), p = 0.77
Gatto et al. (2015) [50]	**0.79 (0.63, 0.98), p = 0.03***	**0.65 (0.47, 0.91), p = 0.01***	1.19 (0.77, 1.84), p 0.41	**1.29 (1.03, 1.63), p = 0.02***
Kang et al. (2016)[ii] [52]	0.83, (0.42, 1.63), p = 0.60	0.71 (0.35, 1.47), p = 0.36	0.23 (0.01, 4.93), p 0.35	1.19 (0.61, 2.29), p = 0.60
Meamar et al. (2016) [53]	1.03 (0.58, 1.81), p = 0.90	1.38 (0.36, 5.20), p = 0.62	1.17 (0.55, 2.48), p = 0.66	1.03 (0.58, 1.84), p = 0.90
Gezen-Ak et al. (2017) [55]	0.93 (0.73, 1.18), p = 0.58	0.81 (0.58, 1.12), p = 0.22	0.84 (0.51, 1.35), p = 0.47	1.06 (0.84, 1.35), p = 0.58
Tanaka et al. (2017)[ii] [56]	0.91 (0.63, 1.31), p = 0.63	0.89 (0.59, 1.34), p = 0.59	1.04 (0.29, 3.72), p =0.95	1.09 (0.76, 1.56), p = 0.62
Agliardi et al. (2021) [58]	0.94 (0.79, 1.11), p = 0.49	0.87 (0.68, 1.12), p = 0.30	0.99 (0.71, 1.37), p = 0.96	1.06 (0.89, 1.26), p = 0.49
Redenšek et al. (2022)[ii] [60]	1.05 (0.78, 1.40), p = 0.76	0.96 (0.63, 1.45), p = 0.85	0.78 (0.45, 1.35), p = 0.38	0.95 (0.71, 1.27), p = 0.73
Canales-Cortés et al. (2024) [61]	0.58 (0.23, 1.48), p = 0.25	0.48 (0.15, 1.50), p = 0.20	1.53 (0.16, 14.14), p = 0.70	1.73 (0.67, 4.49), p = 0.25
Kundu et al. (2025) [62]	0.68 (0.44, 1.06), p = 0.09	**0.50 (0.28, 0.88), p = 0.01***	0.65 (0.17, 2.38), p = 0.51	1.55 (0.95, 2.50), p = 0.07
Overall	1.02 (0.93, 1.11), p = 0.64	0.95 (0.86, 1.05), p = 0.36	0.94 (0.80, 1.12), p = 0.53	1.01 (0.94, 1.09), p = 0.68
**Tru9I (rs757343)**				

Gezen-Ak et al. (2017) [55]	1.21 (0.91, 1.60), p = 0.17	1.23 (0.87, 1.72), p = 0.22	0.71 (0.34, 1.48), p = 0.89	0.82 (0.62, 1.09), p = 0.18
**rs1989969**				
Agliardi et al. (2021) [58]	0.96 (0.81, 1.15), p = 0.70	0.77 (0.54, 1.10), p = 0.15	0.94 (0.73, 1.20), p = 0.62	1.03 (0.87, 1.22), p = 0.70
**rs2853559**				
Lin et al. (2014)[ii] [49]	1.09 (0.94, 1.27), p = 0.24	1.17 (0.95, 1.44), p = 0.12	0.98 (0.72, 1.35), p = 0.93	0.91 (0.78, 1.06), p = 0.24
**rs4334089**				
Lv et al. (2013) [47]	1.01 (0.85, 1.21), p = 0.86	0.95 (0.73, 1.25), p = 0.76	0.89 (0.65, 1.23), p = 0.50	0.98 (0.82, 1.17), p = 0.86
Lin et al. (2014)[ii] [49]	1.03 (0.89, 1.19), p = 0.63	1.04 (0.83, 1.29), p = 0.70	0.94 (0.71, 1.23), p = 0.66	0.96 (0.82, 1.11), 0.61
Gatto et al. (2015) [50]	1.01 (0.79, 1.29), p = 0.91	0.99 (0.73, 1.34), p = 0.96	0.87 (0.46, 1.66), p = 0.69	0.98 (0.76, 1.26), p = 0.90
Fazeli et al. (2016) [51]	**0.79 (0.67, 0.95), p = 0.01***	0.83 (0.62, 1.10), p = 0.20	**1.49 (1.13, 1.96), p < 0.01***	**1.24 (1.05, 1.48), p = 0.01***
Overall	**0.86 (0.77, 0.96), p < 0.01***	0.90 (0.79, 1.03), p = 0.13	1.14 (0.98, 1.34), p = 0.08	1.08 (0.99, 1.18), p = 0.06
**rs7299460**				
Lin et al. (2014)[ii] [49]	1.01 (0.88, 1.17), p = 0.82	1.02 (0.77, 1.35), p = 0.85	0.97 (0.78, 1.20), p = 0.81	0.98 (0.84, 1.13), p = 0.79
**rs7968585**				
Lin et al. (2014)[ii] [49]	1.00 (0.85, 1.31), p = 1.00	0.98 (0.80, 1.20), p = 0.86	0.94 (0.65, 1.34), p = 0.73	0.99 (0.85, 1.16), p = 0.98
**rs7976091**				
Török et al. (2013) [48]	0.84 (0.57, 1.26), p = 0.81	0.73 (0.35, 1.52), p = 0.41	1.15 (0.66, 2.00), p = 0.60	1.16 (0.79, 1.69), p = 0.43
Lin et al. (2014)[ii] [49]	1.10 (0.95, 1.27), p = 0.20	1.08 (0.87, 1.35), p = 0.44	0.81 (0.61, 1.07), p = 0.14	0.90 (0.77, 1.05), p = 0.18
Kang et al. (2016)[ii] [52]	0.85 (0.62, 1.18), p = 0.36	0.63 (0.38, 1.04), p = 0.07	0.84 ((0.46, 1.55), p = 0.59	**2.24 (1.68, 3.00), p < 0.01***
Mohammadzadeh et al. (2016) [54]	**12.66 (7.38, 21.71), p < 0.01***	**6.41 (2.58, 15.91), p < 0.01***	**0.07 (0.04, 0.13), p < 0.01***	**0.74 (0.57, 0.97), p = 0.03***
Canales-Cortés et al. (2024) [61]	0.64 (0.29, 1.42), p = 0.28	0.68 (0.18, 2.56), p = 0.57	2.69 (0.54, 13.29), p = 0.22	**5.39 (2.23, 12.97), p < 0.01***
Overall	**2.09 (1.59, 2.75), p < 0.01***	1.05 (0.88, 1.26), p = 0.56	**0.62 (0.51, 0.75), p < 0.01***	**0.83 (0.74, 0.93), p < 0.01***
**rs10083198**				

Lin et al. (2014)[a] [49]	0.90 (0.78, 1.05), p = 0.20	0.95 (0.76, 1.19), p = 0.71	1.16 (0.90, 1.50), p = 0.24	**2.05 (1.80, 2.32) p < 0.01***

* significant results with $p < 0.05$

Notes:

i) ■, dominant allele (e.g., A); ○, recessive allele (e.g., a)

Allele: ■vs○ = A vs a

Dominant: ■■vs■○+○○ = AA vs. Aa +aa

Recessive:○○vs■■+■○ = aa vs AA +Aa

Additive: ■■vs■○vs○○ = AA vs. Aa vs. aa

ii) For those without isolated allele count, the formula below was used: Count Alleles: Each individual contributes two alleles. For genotype AA, both alleles are A. For Aa, one A and one a. For aa, both alleles are a. So for each group: A alleles = (2 × AA) + (1 × Aa); a alleles = (2 × aa) + (1 × Aa).

TABLE S14

Table S14. Effects of VitD in In Vitro Models of PD.

PD model	VitD form & dose	Observed effect	Reference
C6 glioma cells	1α,25$(OH)_2D_3$ (10^{-8} M)	↑M-CSF & LIF mRNA; ↑by LPS-synergistic effect; ↔TNF-α expression	Furman et al. (1996) [65]
Primary rat astrocytes		↓LPS-induced M-CSF and TNF-α mRNA (~30%); ↔on LIF mRNA	
Cultured BAMC	1α,25$(OH)_2D_3$ (0.1–100 nM)	↑TH mRNA (2–3x); ↔nicotine; ↔Ca^{2+} antagonist TMB-8; suggests Ca^{2+}-independent	Puchacz et al. (1996) [66]
L929 mouse fibroblasts	1α,25$(OH)_2D_3$ (0.01–10 nM); EB-1089 & OCT (1 nM)	↑NGF synthesis (2–3x); ↑ NGF synthesis (6x) with ↑VDR via forskolin; EB-1089 & OCT equally potent	Musiol et al. (1997) [67]
Rat primary astrocytes (LPS-induced inflammation)	1α,25$(OH)_2D_3$ (10^{-8} to 10^{-6} M)	↑γ-GT mRNA & activity; ↑GSH levels; ↓NO_2^- accumulation; ↔SOD or GSH peroxidase expression	Garcion et al. (1999) [68]
Rat mesencephalic neurons exposed to BSO and MPP^+	Calcitriol (1–100 nM)	↑neuron survival and neurite growth; ↓ROS & GSH depletion	Shinpo et al. (2000) [69]
Rat mesencephalic cultures exposed to glutamate, 6-OHDA, and MPP^+	Calcitriol (10–100 nM)	↑cell viability	Ibi et al. (2001) [70]
Primary cultures of rat ventral mesencephalon treated with H_2O_2 and 6-OHDA	Calcitriol (0.1 nM) on day 7 before toxin exposure	↑cell survival	Wang et al. (2001) [71]

https://doi.org/10.3390/neurosci6040130

Neonatal rat brain (SVZ neurosphere assay)	VitD maternal depletion; 1α,25(OH)$_2$D$_3$ (100 nM in vitro)	Maternal VitD deficiency → ↑neurospheres (↑ proliferation); VitD → ↓ neurosphere, but not DVD cultures; VDR expressed in SVZ & neurospheres	Cui et al. (2007) [72]
Rat fetal ventral mesencephalic dopamine neuron cultures	Calcitriol (100 pM–100 nM) for 7 days	↑dopamine neuron count & GDNF expression; ↓apoptosis	Orme et al. (2013) [73]
SH-SY5Y cells exposed to rotenone	Calcitriol (2.5–10 μM)	↓ROS; ↑survival-related signaling proteins and autophagy markers (LC3, beclin-1, AMPK)	Jang et al. (2014) [74]
Neural stem cells injured by L-DOPA	Calcitriol (10–1000 nM)	↓free radical levels; boosted cell viability, proliferation, and PI3K pathway activation	Jang et al. (2015) [75]
SH-SY5Y cells with VDR overexpression	1,25(OH)$_2$D$_3$ (20 nM)	↑neuronal development and maturation; ↑TH, COMT, MAO-A, VMAT2 expression	Pertile et al. (2016) [76]
HN9.10e embryonic hippocampal cells	Vitamin D3 (100 nM for 48 h)	↑nSMase activity; ↓saturated SM species; ↑unsaturated SM species	Cataldi et al. (2017) [77]
SH-SY5Y human neuroblastoma cells	Calcipotriol (0.1–10 nM)	↓αSyn aggregation; ↑cell viability	Rcom-H'cheo-Gauthier et al. (2017) [78]
HN9.10e embryonic hippocampal cells	Vitamin D3 (100 nM for 48 h)	↑VDR expression; ↑MAP2 & NEFH expression; ↑NF200 & N-cadherin proteins; neurite outgrowth and differentiation	Cataldi et al. (2018) [79]
SH-SY5Y neuroblastoma cells transfected with VDR	Calcitriol (20 nM)	↑dopaminergic differentiation & survival; ↑C-Ret, GDNF, GFRα1 expression	Pertile et al. (2018) [80]
MPP$^+$-induced PD cell model (SH-SY5Y)	Calcitriol (25–75 nM)	Protected cells from parthanatos; ↓ PARP1, AIF, phospho-H2A.X	Hu et al. (2021) [81]
SH-SY5Y cells treated with αSyn oligomers	Vitamin D (4 μM) for 36 h	↑ cell viability; ↓αSyn aggregation, cytotoxicity, and ROS	Zhang et al. (2022) [82]
Rotenone-induced PD model in PC12 cells	1α,25(OH)$_2$D$_3$ (0.1–1.0 ng/mL)	↑ cell viability; ↓ ROS and SOD; ↑ GSH; ↑ TH, Nrf2, VD3R; ↓ NF-κB; restored mitochondrial potential	de Siqueira et al. (2023) [83]

Abbreviations: AIF, Apoptosis-inducing factor; AMPK, AMP-activated protein kinase; BAMC, Bovine adrenal medullary cells; BSO, Buthionine sulfoximine; C-Ret, Proto-oncogene tyrosine-protein kinase receptor Ret; COMT, Catechol-O-methyltransferase; Ca^{2+}, Calcium ion; DVD, Developmental vitamin D deficiency; EB-1089, Vitamin D analog EB-1089; GDNF, Glial cell line-derived neurotrophic factor; GFRα1, GDNF family receptor alpha-1; GSH, Glutathione; LC3, Microtubule-associated protein 1A/1B-light chain 3; LIF, Leukemia inhibitory factor; LPS, Lipopolysaccharide; M-CSF, Macrophage colony-stimulating factor; MAO-A, Monoamine oxidase A; MAP2, Microtubule-associated protein 2; MPP$^+$, 1-Methyl-4-phenylpyridinium; NEFH, Neurofilament heavy polypeptide; NF-κB, Nuclear factor kappa-light-chain-enhancer of activated B cells; NF200, Neurofilament 200; NGF, Nerve growth factor; NO_2^-, Nitrite; OCT, Vitamin D analog OCT; PARP1, Poly (ADP-ribose) polymerase 1; PC12, Rat pheochromocytoma cells; PI3K, Phosphoinositide 3-kinase; ROS, Reactive oxygen species; SOD, Superoxide dismutase; SVZ, Subventricular zone; TH, Tyrosine hydroxylase; TMB-

https://doi.org/10.3390/neurosci6040130

8, Calcium antagonist TMB-8; TNF-α, Tumor necrosis factor alpha; VD3R, Vitamin D3 receptor; VDR, Vitamin D receptor; VMAT2, Vesicular monoamine transporter 2; nSMase, Neutral sphingomyelinase; αSyn, Alpha-synuclein; γ-GT, Gamma-glutamyltransferase. ↑, increase/ upregulate; ↓, decrease/ downregulate; ↔, no effect, no dependent, unclear.

https://doi.org/10.3390/neurosci6040130

TABLE S15

Table S15. Effects of VitD in In Vivo Models of PD.			
PD model	VitD form & dose	Observed effect	Reference
Rat model with 6-OHDA-induced neurotoxicity	Calcitriol (1 μg/mL, 1 mL/kg, i.p. daily for 8 days)	↑locomotor performance; ↑striatal dopamine, DOPAC, and HVA levels	Wang et al. (2001) [71]
Adult rat model (no lesion)	1,25-dihydroxyvitamin D_3 (1 μg/kg, i.p. daily for 7 days)	↑GDNF mRNA & protein levels in striatum, suggesting neurotrophic support for dopaminergic neurons	Sanchez et al. (2002) [84]
Rat model with intranigral zinc infusion (20 nmoles)	Calcitriol (Vitamin D3, 1 μg/mL/kg/day, i.p. for 13 days)	↓lipid peroxidation; ↓cytosolic cytochrome c; ↑striatal dopamine levels	Lin et al. (2003) [85]
VDR knockout mice	Genetic deletion (VDR−/−)	Severe motor impairment in vertical screen and swim tests; ↔sensory or emotional deficits; linked to ↓calcium & weakness	Kalueff et al. (2004) [86]
VDR knockout mice	Genetic deletion (VDR−/−)	↓locomotor activity, impaired motor coordination, ↓habituation, ↓ PPI at long intervals; ↔cognitive or anxiety-related deficits	Burne et al. (2005) [87]
Rat and mouse models (6-OHDA and MPTP)	1,25-dihydroxyvitamin D_3 (1 μg/mL, 1 mL/kg, i.p. daily for 7 days)	↓dopaminergic neuron loss; ↓microglial activation; ↓TNF-α & IFN-γ expression	Kim et al. (2006) [88]
ICV 6-OHDA PD rat model	Calcitriol (1.0 μg/kg/day, short vs. long-term)	Long-term: ↑DA overflow & striatal DA content; ↑GDNF in SN; Short-term: no protection despite ↑GDNF in SN	Smith et al. (2006) [89]
Rat model with 6-OHDA lesion in medial forebrain bundle	1,25-Dihydroxyvitamin D3 (Calcitriol, 1 μg/mL/kg/day, i.p. for 7 days pre- or post-lesion)	↑GDNF protein in striatum and SN; partial restoration of TH expression in SN and striatum	Sanchez et al. (2009) [90]
Developmental vitamin D-deficient rat model (chronic)	Calcitriol (0 IU/kg) in embryonic forebrain cultures (E18, Sprague-Dawley rats)	↓differentiation & maintenance of dopaminergic neurons; ↓ C-Ret expression	Cui et al. (2010) [91]
Normal rats	Calcitriol (1.0–3.0 μg/kg/day, s.c.)	↑Stimulus-evoked DA overflow in striatum; ↑DA and metabolites in substantia nigra; ↑GDNF in striatum and SN; ↔basal DA	Cass et al. (2012) [92]
Mouse model with MPTP-induced neurotoxicity	25-Hydroxyvitamin D depletion (via vitamin D-deficient diet for 6 weeks)	↔MPTP-induced DA damage; ↔DA, TH, DAT, or behavior	Dean et al. (2012) [93]

https://doi.org/10.3390/neurosci6040130

6-OHDA-lesioned rat model	Vitamin D3, 1 μg/kg twice a week for 4 weeks	↑VEGF in striatum, neuroprotective effect against 6-OHDA-induced damage	Hashemvarzi et al. (2012) [94]
Rat model with repeated haloperidol administration	Endogenous 1,25-dihydroxyvitamin D_3 (no supplementation)	↔VitD signaling (cyp27b1, cyp24a1, VDR); ↑Nur77, RXRβ, and RXRγ expression in prefrontal cortex	Jiang et al. (2013) [95]
6-OHDA-lesioned rats	Calcitriol (0.3–1.0 μg/kg/day, s.c.)	↑DA release & tissue levels in striatum & substantia nigra; partial restoration of DA function; ↑GDNF (minor)	Cass et al. (2014) [96]
MPTP mouse model	1,25-dihydroxyvitamin D_3 (calcitriol), 0.2–5 μg/kg/day orally	Neuroprotection via ↑TH, ↓αSyn, and autophagy induction (↑LC3-II, Beclin-1; ↓mTOR, p62)	Li et al. (2015) [97]
C. elegans (aging model)	Vitamin D3 (25–250 μM)	↓insoluble protein accumulation; ↑lifespan	Mark et al. (2016) [98]
MPTP-induced acute PD model in mice	Vitamin D (1 μg/kg/day for 10 days)	↓neuroinflammation. Modulation of iNOS, TLR-4, IL-10, IL-4, TGF-β, CD163, CD206, CD204	Calvello et al. (2017) [99]
Age-stratified rat model (4, 14, and 22 months) with 6-OHDA lesion	Calcitriol (1 μg/kg, s.c., daily for 8 days, starting 4 weeks post-lesion)	↑DA overflow in the striatum of young & middle-aged rats; ↑dopamine in substantia nigra across all age groups	Cass et al. (2017) [100]
MPTP-induced PD mouse model	Endogenous modulation (no external dose specified)	↓nSMase expression; ↑iNOS in hippocampus dentate gyrus	Cataldi et al. (2017) [77]
MPTP-induced PD mouse model	Endogenous modulation (no external dose specified)	↓VDR & GFAP expression in dentate gyrus of hippocampus; suggests impaired neurogenesis	Cataldi et al. (2018) [79]
Rat model with 6-OHDA lesion	Calcitriol (1 μg/kg/day, oral) for 7 days before or 14 days after lesion	↓behavioral deficits; DA depletion; ↑mitochondrial function; ↑TH, DAT, VDAC, and Hsp60 expression	Lima et al. (2018) [101]
Mouse model with 6-OHDA lesion	Calcitriol (2.56 μg/kg, i.p., every other day, 4 doses total)	↓DA neuron loss; ↓neuroinflammation; ↑VDR; ↑ endothelial P-glycoprotein	Kim et al. (2020) [102]
6-OHDA-induced acute PD model in mice	$1,25(OH)_2D_3$ (2.56 μg/kg, every other day × 4 doses)	↓αSyn aggregation & toxicity; ↑P-glycoprotein expression	Kim et al. (2020) [102]
In vitro MPP^+-induced neurotoxicity model	VDR antagonist ZAV-12 (10–20 μM)	↑LC3 lipidation; ↑autophagy; ↓αSyn oligomerization & neurotoxicity	Ding et al. (2021) [103]
MPTP-induced subacute PD model in mice	Calcitriol (2.5 μg/kg/day, i.p., for 21 days)	↑motor performance in rotarod & pole tests; ↓PD-like behavioral impairments	Hu et al. (2021) [81]

https://doi.org/10.3390/neurosci6040130

Rat model with 6-OHDA striatal lesion	Vitamin D3 (1 μg/kg/day, oral, pre- or post-lesion)	↑mitochondrial function (oxygen consumption, RCR, ADP/O); ↓H_2O_2; ↑SOD; ↑TH/DAT; ↓VDAC1/ Hsp60	Araújo de Lima et al. (2022) [104]
Mouse model with 6-OHDA lesion	Cholecalciferol ± L-DOPA, administered 2 weeks post-lesion	↑motor & cognitive behavior; ↓oxidative stress & DA metabolism markers; ↑TH, DAT, and BDNF; ↓MAO-B, CD11b, IL-1β, and p47phox	Bayo-Olugbami et al. (2022) [105]
Rotenone-induced PD in rat	Calcitriol (1 μg/kg/day, i.p., for 4 weeks; prophylactic and post-treatment)	↑motor performance and coordination; ↑TH expression; ↑Sirt1 & LC3; ↓P62 & NF-κB; improved autophagy & histopathology	Magdy et al. (2022) [106]
Rat model with 6-OHDA lesion and treadmill exercise	Cholecalciferol (1 μg/kg/day for 21 days) ± physical activity (30 min/day)	↑behavioral recovery; ↑DA, DOPAC, TH, DAT, and VDR; ↓oxidative stress and nitrite levels	da Costa et al. (2023) [107]
Rat model with lead-induced SNpc toxicity	Vitamin D3 (1,000 IU/kg, i.m., 3×/week for 8 weeks)	↓αSyn and caspase-11; ↑ TH and oligodendrocyte markers; ↑neuronal density and morphology	Imam et al. (2023) [108]
Female mice and fetuses	1,25-dihydroxyvitamin D3, 1 μg/ml × 1 ml/kg/day for 21 days	↑neuronal viability; ↓apoptosis & necrosis; modulated gene expression	Khosravi et al. (2023) [109]

Abbreviations: αSyn, Alpha-synuclein; 6-OHDA, 6-Hydroxydopamine; ADP/O, Adenosine diphosphate to oxygen ratio; BDNF, Brain-derived neurotrophic factor; C-Ret, Proto-oncogene tyrosine-protein kinase receptor Ret; CD11b, Cluster of Differentiation 11b; CD163, Cluster of Differentiation 163; CD204, Cluster of Differentiation 204; CD206, Cluster of Differentiation 206; DA, Dopamine; DAT, Dopamine transporter; DOPAC, 3,4-Dihydroxyphenylacetic acid; GDNF, Glial cell line-derived neurotrophic factor; GFAP, Glial fibrillary acidic protein; H_2O_2, Hydrogen peroxide; Hsp60, Heat shock protein 60; HVA, Homovanillic acid; ICV, Intracerebroventricular; IFN-γ, Interferon gamma; IL-1β, Interleukin 1 beta; IL-4, Interleukin 4; IL-10, Interleukin 10; iNOS, Inducible nitric oxide synthase; IU, International Units; L-DOPA, Levodopa; LC3, Microtubule-associated protein 1A/1B-light chain 3; LC3-II, Lipidated form of LC3; MAO-B, Monoamine oxidase B; MPP^+, 1-Methyl-4-phenylpyridinium; MPTP, 1-Methyl-4-phenyl-1,2,3,6-tetrahydropyridine; mTOR, Mechanistic target of rapamycin; nSMase, Neutral sphingomyelinase; NF-κB, Nuclear factor kappa-light-chain-enhancer of activated B cells; Nur77, Nuclear receptor subfamily 4 group A member 1; PPI, Prepulse inhibition; RCR, Respiratory control ratio; RXRβ, Retinoid X receptor beta; RXRγ, Retinoid X receptor gamma; SN, Substantia nigra; SNpc, Substantia nigra pars compacta; SOD, Superoxide dismutase; TH, Tyrosine hydroxylase; TLR-4, Toll-like receptor 4; TNF-α, Tumor necrosis factor alpha; VDAC, Voltage-dependent anion channel; VDAC1, Voltage-dependent anion channel 1; VDR, Vitamin D receptor.↑, increase/ upregulate; ↓, decrease/ downregulate; ↔, no effect, no dependent, unclear.

TABLE S16

Table S16. Amounts of Vitamin D and Energy per Standard Portion.

Food item	Portions (standard)	Calories	Vitamin D (IU)	% Daily values*
Mushrooms (UV-exposed, raw)	1 cup	~15–20	0–1110	111.0%
Rainbow trout (freshwater)	3 oz	142	645	64.5%
Salmon (various types)	3 oz	115–175	383–570	57.0%
Light tuna (canned)	3 oz	168	231	23.1%
Herring	3 oz	172	182	18.2%
Sardines (canned)	3 oz	177	164	16.4%
Tilapia	3 oz	108	127	12.7%
Soy milk (unsweetened, fortified)	1 cup	80	119	11.9%
Flounder	3 oz	73	118	11.8%
Milk (low fat, 1%)	1 cup	102	117	11.7%
Yogurt (plain, nonfat)	8 oz	137	116	11.6%
Yogurt (plain, low fat)	8 oz	154	116	11.6%
Milk (fat-free/skim)	1 cup	83	115	11.5%
Almond milk (unsweetened, fortified)	1 cup	36	107	10.7%
Rice milk (unsweetened, fortified)	1 cup	113	101	10.1%
Kefir (plain, low fat)	1 cup	104	100	10.0%
Orange juice (100%, fortified)	1 cup	117	100	10.0%
Cheese (American, fortified)	1.5 oz	104	85	8.5%

Abbreviation: IU, International Units

* RDA(1000 IU per day)

Source: https://www.dietaryguidelines.gov/resources/2020-2025-dietary-guidelines-online-materials/food-sources-select-nutrients/food-sources-vitamin-d, accessed on 8/29/2025.

TABLE S17

Table S17. Clinical trials assessing the effect of VitD on PD.

ClinicalTrials.gov ID	Primary outcome	Secondary outcome	VitD therapy	Start
NCT07084597	VitD effect on cardiac autonomic dysfunction (HRV)	None	50K IU/week for 8 wks	Jul, 2025
NCT07096336	VitD effect on quantitative EEG	None	50K IU/week for 8 wks	Jul, 2025
NCT06697626	VitD effect on motor and non motor symptoms (UPDRS, BSC, GSRS, PDQ39, gut microbiome)	VitD, inflammation (CRP, IL-6, IL-10, TNF-α)	Functional drink with VitD 5μg BID for 12 wks	Nov, 2024
NCT06539260	VitD effect on Treg and Th17	UPDRS, Berg, MMSE, MoCA, SDS, SAS	400 IU BID for 12 wks	Jan, 2023
NCT04768023	VitD effect on physical activity and balance (6MWT, 10MWT, TUG); inflammation	None	4–6K IU/day for 12 wks	Nov, 2019
NCT00907972	VitD effect on bone mass density	UPDRS, PD QoL, and falls	1K IU/d for 1 year	Sept, 2013
NCT02110875	VitD effect on balance (SOT) and strength (Biodex)	None	10K IU/d and calcium 1K mg/d for 16 wks	Jun, 2013
NCT01119131	VitD effect on balance (SOT, TUG)	TMT, PDQ39, UPDRS	10K IU/d and calcium 1K mg/d for 16 wks	May, 2011
NCT00571285	Vit effect on motor symptoms (UPDRS-III and TUG)	UPDRS-II, BAI-II, BD-II	50K IU/week and 1K IU/d for 26 wks	Jun, 2007

Abbreviations: 6MWT, 6 minute walk test; 10MWT, 10 minute walk test; BAI-II, Beck Anxiety Inventory; BD-II, Beck Depression Inventory;Berg, Berg balance scale; BID, bis in die, twice a day; BSC, Bristol Stool Chart; CRP, C-reactive protein; d, day; GSRS, Gastrointestinal Symptom Rating Scale; HRV, heart rate variability; IL-6, interleukin 6; IL-10, interleukin 10; MMSE, Mini Mental State Examination; MoCA, Montreal Cognitive Assessment; PD, Parkinson's disease; PDQ39, Parkinson's Disease Questionnaire; QoL, quality of life; SDS, Zung Self-Rating Depression Scale; SAS, Zung Self-Rating Anxiety Scale; SOT, Sensory Organization Test; TMT, Trail Making Test; TNF-α, tumour necrosis factor; TUG, Timed Up and Go; UPDRS, Unified Parkinson Disease Rating Scale; VitD, vitamin D; wk, week

Functional drink: 10g chicory inulin 100μg folic acid 5μg vitamin B12 5mg vitamin B6 2.5mg riboflavin 5μg vitamin D

References

1. Abou-Raya, S.; Helmii, M.; Abou-Raya, A. Bone and Mineral Metabolism in Older Adults with Parkinson's Disease. *Age Ageing* **2009**, *38*, 675–680, doi:10.1093/ageing/afp137.

2. Evatt, M.L.; Delong, M.R.; Khazai, N.; Rosen, A.; Triche, S.; Tangpricha, V. Prevalence of Vitamin d Insufficiency in Patients with Parkinson Disease and Alzheimer Disease. *Arch Neurol* **2008**, *65*, 1348–1352, doi:10.1001/archneur.65.10.1348.

3. van den Bos, F.; Speelman, A.D.; van Nimwegen, M.; van der Schouw, Y.T.; Backx, F.J.G.; Bloem, B.R.; Munneke, M.; Verhaar, H.J.J. Bone Mineral Density and Vitamin D Status in Parkinson's Disease Patients. *J Neurol* **2013**, *260*, 754–760, doi:10.1007/s00415-012-6697-x.

4. Serdaroğlu Beyazal, M.; Kırbaş, S.; Tüfekçi, A.; Devrimsel, G.; Küçükali Türkyılmaz, A. The Relationship of Vitamin D with Bone Mineral Density in Parkinson's Disease Patients. *European Geriatric Medicine* **2016**, *7*, 18–22, doi:10.1016/j.eurger.2015.08.002.

5. Yoon, J.H.; Park, D.K.; Yong, S.W.; Hong, J.M. Vitamin D Deficiency and Its Relationship with Endothelial Dysfunction in Patients with Early Parkinson's Disease. *J Neural Transm (Vienna)* **2015**, *122*, 1685–1691, doi:10.1007/s00702-015-1452-y.

6. Wang, J.; Yang, D.; Yu, Y.; Shao, G.; Wang, Q. Vitamin D and Sunlight Exposure in Newly-Diagnosed Parkinson's Disease. *Nutrients* **2016**, *8*, 142, doi:10.3390/nu8030142.

7. Ding, H.; Dhima, K.; Lockhart, K.C.; Locascio, J.J.; Hoesing, A.N.; Duong, K.; Trisini-Lipsanopoulos, A.; Hayes, M.T.; Sohur, U.S.; Wills, A.-M.; et al. Unrecognized Vitamin D3 Deficiency Is Common in Parkinson Disease: Harvard Biomarker Study. *Neurology* **2013**, *81*, 1531–1537, doi:10.1212/WNL.0b013e3182a95818.

8. Wang, L.; Evatt, M.L.; Maldonado, L.G.; Perry, W.R.; Ritchie, J.C.; Beecham, G.W.; Martin, E.R.; Haines, J.L.; Pericak-Vance, M.A.; Vance, J.M.; et al. Vitamin D from Different Sources Is Inversely Associated with Parkinson Disease. *Mov Disord* **2015**, *30*, 560–566, doi:10.1002/mds.26117.

9. Knekt, P.; Kilkkinen, A.; Rissanen, H.; Marniemi, J.; Sääksjärvi, K.; Heliövaara, M. Serum Vitamin D and the Risk of Parkinson Disease. *Arch Neurol* **2010**, *67*, 808–811, doi:10.1001/archneurol.2010.120.

10. Topal, K.; Paker, N.; Bugdayci, D.; Ozer, F.; Tekdos, D. Bone Mineral Density and Vitamin D Status with Idiopathic Parkinson's Disease.; SPRINGER LONDON LTD 236 GRAYS INN RD, 6TH FLOOR, LONDON WC1X 8HL, ENGLAND, 2010; Vol. 21, pp. 141–142.

11. Senel, K.; Alp, F.; Baykal, T.; Melikoglu, M.; Erdal, A.; Ugur, M. Preliminary Study: Is There a Role of Vitamin D in Parkinson Disease?; SPRINGER LONDON LTD 236 GRAYS INN RD, 6TH FLOOR, LONDON WC1X 8HL, ENGLAND, 2011; Vol. 22, pp. 167–167.

12. Meamar, R.; Maracy, M.; Chitsaz, A.; Ghazvini, M.R.A.; Izadi, M.; Tanhaei, A.P. Association between Serum Biochemical Levels, Related to Bone Metabolism and Parkinson's Disease. *J Res Med Sci* **2013**, *18*, S39-42.

13. Petersen, M.S.; Bech, S.; Christiansen, D.H.; Schmedes, A.V.; Halling, J. The Role of Vitamin D Levels and Vitamin D Receptor Polymorphism on Parkinson's Disease in the Faroe Islands. *Neurosci Lett* **2014**, *561*, 74–79, doi:10.1016/j.neulet.2013.12.053.

14. Liu, Y.; Zhang, B.-S. Serum 25-Hydroxyvitamin D Predicts Severity in Parkinson's Disease Patients. *Neurol Sci* **2014**, *35*, 67–71, doi:10.1007/s10072-013-1539-x.

15. Ozturk, E.A.; Gundogdu, I.; Tonuk, B.; Kocer, B.G.; Tombak, Y.; Comoglu, S.; Cakci, A. Bone Mass and Vitamin D Levels in Parkinson's Disease: Is There Any Difference between Genders? *J Phys Ther Sci* **2016**, *28*, 2204–2209, doi:10.1589/jpts.28.2204.

16. Hatem, A.K. The State of Vitamin D in Iraqi Patients With Parkinson Disease. *Al-Kindy Col. Med. J* **2019**, *13*, 137–141, doi:10.47723/kcmj.v13i1.144.

17. Sleeman, I.; Aspray, T.; Lawson, R.; Coleman, S.; Duncan, G.; Khoo, T.K.; Schoenmakers, I.; Rochester, L.; Burn, D.; Yarnall, A. The Role of Vitamin D in Disease Progression in Early Parkinson's Disease. *J Parkinsons Dis* **2017**, *7*, 669–675, doi:10.3233/JPD-171122.

18. Ahangar, A.A.; Saadat, P.; Hajian, K.; Kiapasha, G. The Association between Low Levels of Serum Vitamin D and the Duration and Severity of Parkinson's Disease. *Arch Neurosci* **2018**, *5*, e61085.

19. Mollenhauer, B.; Zimmermann, J.; Sixel-Döring, F.; Focke, N.K.; Wicke, T.; Ebentheuer, J.; Schaumburg, M.; Lang, E.; Friede, T.; Trenkwalder, C. Baseline Predictors for Progression 4 Years after Parkinson's Disease Diagnosis in the De Novo Parkinson Cohort (DeNoPa). *Mov Disord* **2019**, *34*, 67–77, doi:10.1002/mds.27492.

20. Soliman, R.H.; Oraby, M.I.; Hussein, M.; Abd El-Shafy, S.; Mostafa, S. Could Vitamin D Deficiency Have an Impact on Motor and Cognitive Function in Parkinson's Disease? *The Egyptian Journal of Neurology, Psychiatry and Neurosurgery* **2019**, *55*, 34, doi:10.1186/s41983-019-0084-9.

21. Zhang, H.-J.; Zhang, J.-R.; Mao, C.-J.; Li, K.; Wang, F.; Chen, J.; Liu, C.-F. Relationship between 25-Hydroxyvitamin D, Bone Density, and Parkinson's Disease Symptoms. *Acta Neurol Scand* **2019**, *140*, 274–280, doi:10.1111/ane.13141.

22. Fahmy, E.M.; Elawady, M.E.; Sharaf, S.; Heneidy, S.; Ismail, R.S. Vitamin D Status in Idiopathic Parkinson's Disease: An Egyptian Study. *The Egyptian Journal of Neurology, Psychiatry and Neurosurgery* **2020**, *56*, 45.

23. Ogura, H.; Hatip-Al-Khatib, I.; Suenaga, M.; Hatip, F.B.; Mishima, T.; Fujioka, S.; Ouma, S.; Matsunaga, Y.; Tsuboi, Y. Circulatory 25(OH)D and 1,25(OH)(2)D as Differential Biomarkers between Multiple System Atrophy and Parkinson's Disease Patients. *eNeurologicalSci* **2021**, *25*, 100369, doi:10.1016/j.ensci.2021.100369.
24. Barichella, M.; Cereda, E.; Iorio, L.; Pinelli, G.; Ferri, V.; Cassani, E.; Bolliri, C.; Caronni, S.; Pusani, C.; Schiaffino, M.G.; et al. Clinical Correlates of Serum 25-Hydroxyvitamin D in Parkinson's Disease. *Nutr Neurosci* **2022**, *25*, 1128–1136, doi:10.1080/1028415X.2020.1840117.
25. Kakimoto, A.; Ogura, H.; Suenaga, M.; Mishima, T.; Fujioka, S.; Ouma, S.; Matsunaga, Y.; Tsuboi, Y. Role of Cytochrome P450 for Vitamin D Metabolisms in Patients with Neurodegenerative Disorders. *Clin Park Relat Disord* **2022**, *7*, 100162, doi:10.1016/j.prdoa.2022.100162.
26. Novotnij, D.A.; Zhukova, N.G.; Shperling, L.P.; Stolyarova, V.A.; Zhukova, I.A.; Agasheva, A.E.; Shtaimets, S.V.; Druzhinina, O.A.; Shirokikh, I.V. [Vitamin D and other indicators of calcium-phosphorus metabolism as possible predictors of Parkinson's disease]. *Zh Nevrol Psikhiatr Im S S Korsakova* **2022**, *122*, 56–64, doi:10.17116/jnevro202212208156.
27. Wu, H.; Khuram Raza, H.; Li, Z.; Li, Z.; Zu, J.; Xu, C.; Yang, D.; Cui, G. Correlation between Serum 25(OH)D and Cognitive Impairment in Parkinson's Disease. *J Clin Neurosci* **2022**, *100*, 192–195, doi:10.1016/j.jocn.2022.04.015.
28. Yakşi, E.; Yaşar, M.F. Bone Mineral Density and Vitamin D Levels in Parkinson's Disease: A Retrospective Controlled Study. *Northwestern Med J* **2022**, *2*, 51–58, doi:10.54307/NWMJ.2022.66375.
29. Džoljić, E.; Matutinović, M.S.; Stojković, O.; Veličković, J.; Milinković, N.; Kostić, V.; Ignjatović, S. Vitamin D Serum Levels and Vitamin D Receptor Genotype in Patients with Parkinson's Disease. *Neuroscience* **2023**, *533*, 53–62, doi:10.1016/j.neuroscience.2023.10.004.
30. Sooragonda, B.G.; Sridharan, K.; Benjamin, R.N.; Prabhakar, A.T.; Sivadasan, A.; Kapoor, N.; Cherian, K.E.; Jebasingh, F.K.; Aaron, S.; Mathew, V.; et al. Do Bone Mineral Density, Trabecular Bone Score, and Hip Structural Analysis Differ in Indian Men with Parkinson's Disease? A Case-Control Pilot Study from a Tertiary Center in Southern India. *Ann Indian Acad Neurol* **2023**, *26*, 496–501, doi:10.4103/aian.aian_29_23.
31. Xia, M.; Zhou, Q. Correlation between 25-Hydroxy-Vitamin D and Parkinson's Disease. *IBRO Neurosci Rep* **2024**, *16*, 162–167, doi:10.1016/j.ibneur.2023.02.006.
32. Khan, M.S.H.; Mouri, U.K.; Islam, M.R.; Rahman, T.; Rahman, A.; Ahmed, J.U. Relationship of Vitamin D Deficiency with Non-Motor Functions of Parkinson's Disease. *Bangla J Med* **2024**, *35*, 82–87, doi:10.3329/bjm.v35i2.72400.
33. Milanowski, J.; Nuszkiewicz, J.; Lisewska, B.; Lisewski, P.; Szewczyk-Golec, K. Adipokines, Vitamin D, and Selected Inflammatory Biomarkers among Parkinson's Disease Patients with and without Dyskinesia: A Preliminary Examination. *Metabolites* **2024**, *14*, doi:10.3390/metabo14020106.
34. Rahman, A.; Ahmed, M.J.; Russel, A.H.M.; Hossain, M.A.; Huq, M.N.; Khan, S.M.D.; Rahman, M.M.; Paul, B.; Saha, P.K. Association between Serum Vitamin D Level and Parkinson's Disease: Case Control Study in a Tertiary Level Hospital, Bangladesh. *Bangla J Med* **2024**, *35*, 180–186, doi:10.3329/bjm.v53i3.76493.
35. Xu, Y.; Wang, E.; Zhang, Q.; Liu, J.; Luo, W. Vitamin D and Focal Brain Atrophy in PD with Non-Dementia: A VBM Study. *Front Hum Neurosci* **2024**, *18*, 1474148, doi:10.3389/fnhum.2024.1474148.
36. DuBose, S. EFFECTS OF VITAMIN D SUPPLEMENTATION ON MOTOR SYMPTOMS OF PATIENTS WITH PARKINSON'S DISEASE. **2011**.
37. Suzuki, M.; Yoshioka, M.; Hashimoto, M.; Murakami, M.; Noya, M.; Takahashi, D.; Urashima, M. Randomized, Double-Blind, Placebo-Controlled Trial of Vitamin D Supplementation in Parkinson Disease. *Am J Clin Nutr* **2013**, *97*, 1004–1013, doi:10.3945/ajcn.112.051664.
38. Habibi, A.H.; Anamoradi, A.; Shahidi, G.A.; Razmeh, S.; Alizadeh, E.; Kokhedan, K.M. Treatment of Levodopainduced Dyskinesia with Vitamin D: A Randomized, Double-Blind, Placebo-Controlled Trial. *Neurol Int* **2018**, *10*, 7737, doi:10.4081/ni.2018.7737.
39. Hiller, A.L.; Murchison, C.F.; Lobb, B.M.; O'Connor, S.; O'Connor, M.; Quinn, J.F. A Randomized, Controlled Pilot Study of the Effects of Vitamin D Supplementation on Balance in Parkinson's Disease: Does Age Matter? *PLoS One* **2018**, *13*, e0203637, doi:10.1371/journal.pone.0203637.
40. Barichella, M.; Cereda, E.; Pinelli, G.; Iorio, L.; Caroli, D.; Masiero, I.; Ferri, V.; Cassani, E.; Bolliri, C.; Caronni, S.; et al. Muscle-Targeted Nutritional Support for Rehabilitation in Patients with Parkinsonian Syndrome. *Neurology* **2019**, *93*, e485–e496, doi:10.1212/WNL.0000000000007858.
41. Bytowska, Z.K.; Korewo-Labelle, D.; Berezka, P.; Kowalski, K.; Przewłócka, K.; Libionka, W.; Kloc, W.; Kaczor, J.J. Effect of 12-Week BMI-Based Vitamin D(3) Supplementation in Parkinson's Disease with Deep Brain Stimulation on Physical Performance, Inflammation, and Vitamin D Metabolites. *Int J Mol Sci* **2023**, *24*, doi:10.3390/ijms241210200.
42. Zali, A.; Hajyani, S.; Salari, M.; Tajabadi-Ebrahimi, M.; Mortazavian, A.M.; Pakpour, B. Co-Administration of Probiotics and Vitamin D Reduced Disease Severity and Complications in Patients with Parkinson's Disease: A Randomized Controlled Clinical Trial. *Psychopharmacology (Berl)* **2024**, *241*, 1905–1914, doi:10.1007/s00213-024-06606-9.
43. Li, D.; Ma, X.; Zhang, W.; Zhong, P.; Li, M.; Liu, S. Impact of Vitamin D3 Supplementation on Motor Functionality and the Immune Response in Parkinson's Disease Patients with Vitamin D Deficiency. *Sci Rep* **2025**, *15*, 25154, doi:10.1038/s41598-025-10821-5.

44. Kim, J.-S.; Kim, Y.-I.; Song, C.; Yoon, I.; Park, J.-W.; Choi, Y.-B.; Kim, H.-T.; Lee, K.-S. Association of Vitamin D Receptor Gene Polymorphism and Parkinson's Disease in Koreans. *J Korean Med Sci* **2005**, *20*, 495–498, doi:10.3346/jkms.2005.20.3.495.

45. Han, X.; Xue, L.; Li, Y.; Chen, B.; Xie, A. Vitamin D Receptor Gene Polymorphism and Its Association with Parkinson's Disease in Chinese Han Population. *Neurosci Lett* **2012**, *525*, 29–33, doi:10.1016/j.neulet.2012.07.033.

46. Liu, H.; Han, X.; Zheng, X.; Li, Y.; Xie, A. [Association of vitamin D receptor gene polymorphisms with Parkinson disease]. *Zhonghua Yi Xue Yi Chuan Xue Za Zhi* **2013**, *30*, 13–16, doi:10.3760/cma.j.issn.1003-9406.2013.01.004.

47. Lv, Z.; Tang, B.; Sun, Q.; Yan, X.; Guo, J. Association Study between Vitamin d Receptor Gene Polymorphisms and Patients with Parkinson Disease in Chinese Han Population. *Int J Neurosci* **2013**, *123*, 60–64, doi:10.3109/00207454.2012.726669.

48. Török, R.; Török, N.; Szalardy, L.; Plangar, I.; Szolnoki, Z.; Somogyvari, F.; Vecsei, L.; Klivenyi, P. Association of Vitamin D Receptor Gene Polymorphisms and Parkinson's Disease in Hungarians. *Neurosci Lett* **2013**, *551*, 70–74, doi:10.1016/j.neulet.2013.07.014.

49. Lin, C.-H.; Chen, K.-H.; Chen, M.-L.; Lin, H.-I.; Wu, R.-M. Vitamin D Receptor Genetic Variants and Parkinson's Disease in a Taiwanese Population. *Neurobiol Aging* **2014**, *35*, 1212.e11-13, doi:10.1016/j.neurobiolaging.2013.10.094.

50. Gatto, N.M.; Sinsheimer, J.S.; Cockburn, M.; Escobedo, L.A.; Bordelon, Y.; Ritz, B. Vitamin D Receptor Gene Polymorphisms and Parkinson's Disease in a Population with High Ultraviolet Radiation Exposure. *J Neurol Sci* **2015**, *352*, 88–93, doi:10.1016/j.jns.2015.03.043.

51. Fazeli, A.; Motallebi, M.; Jamshidi, J.; Movafagh, A.; Ghaedi, H.; Emamalizadeh, B.; Kashani, K.; Darvish, H. Vitamin D Receptor Gene Rs4334089 Polymorphism and Parkinson's Disease in Iranian Population. *Basal Ganglia* **2016**, *6*, 157–160, doi:10.1016/j.baga.2016.04.001.

52. Kang, S.Y.; Park, S.; Oh, E.; Park, J.; Youn, J.; Kim, J.S.; Kim, J.-U.; Jang, W. Vitamin D Receptor Polymorphisms and Parkinson's Disease in a Korean Population: Revisited. *Neurosci Lett* **2016**, *628*, 230–235, doi:10.1016/j.neulet.2016.06.041.

53. Meamar, R.; Javadirad, S.M.; Chitsaz, N.; Asadian Ghahfarokhi, M.; Kazemi, M.; Ostadsharif, M. Vitamin D Receptor Gene Variants in Parkinson's Disease Patients. *Egyptian Journal of Medical Human Genetics* **2017**, *18*, 225–230, doi:10.1016/j.ejmhg.2016.08.004.

54. Mohammadzadeh, R.; Pazhouhesh, R. Association of VDR FokI and ApaI Genetic Polymorphisms with Parkinson's Disease Risk in South Western Iranian Population. *Acta Medica International* **2016**, *3*.

55. Gezen-Ak, D.; Alaylıoğlu, M.; Genç, G.; Gündüz, A.; Candaş, E.; Bilgiç, B.; Atasoy, İ.L.; Apaydın, H.; Kızıltan, G.; Gürvit, H.; et al. GC and VDR SNPs and Vitamin D Levels in Parkinson's Disease: The Relevance to Clinical Features. *Neuromolecular Med* **2017**, *19*, 24–40, doi:10.1007/s12017-016-8415-9.

56. Tanaka, K.; Miyake, Y.; Fukushima, W.; Kiyohara, C.; Sasaki, S.; Tsuboi, Y.; Oeda, T.; Shimada, H.; Kawamura, N.; Sakae, N.; et al. Vitamin D Receptor Gene Polymorphisms, Smoking, and Risk of Sporadic Parkinson's Disease in Japan. *Neurosci Lett* **2017**, *643*, 97–102, doi:10.1016/j.neulet.2017.02.037.

57. Hu, W.; Wang, L.; Chen, B.; Wang, X. Vitamin D Receptor Rs2228570 Polymorphism and Parkinson's Disease Risk in a Chinese Population. *Neurosci Lett* **2020**, *717*, 134722, doi:10.1016/j.neulet.2019.134722.

58. Agliardi, C.; Guerini, F.R.; Zanzottera, M.; Bolognesi, E.; Meloni, M.; Riboldazzi, G.; Zangaglia, R.; Sturchio, A.; Casali, C.; Di Lorenzo, C.; et al. The VDR FokI (Rs2228570) Polymorphism Is Involved in Parkinson's Disease. *J Neurol Sci* **2021**, *428*, 117606, doi:10.1016/j.jns.2021.117606.

59. Fahmy, E.M.; Elawady, M.E.; Sharaf, S.; Heneidy, S.; Ismail, R.S. Vitamin D Receptor Gene Polymorphisms and Idiopathic Parkinson Disease: An Egyptian Study. *The Egyptian Journal of Neurology, Psychiatry and Neurosurgery* **2021**, *57*, 102, doi:10.1186/s41983-021-00358-5.

60. Redenšek, S.; Kristanc, T.; Blagus, T.; Trošt, M.; Dolžan, V. Genetic Variability of the Vitamin D Receptor Affects Susceptibility to Parkinson's Disease and Dopaminergic Treatment Adverse Events. *Front Aging Neurosci* **2022**, *14*, 853277, doi:10.3389/fnagi.2022.853277.

61. Canales-Cortés, S.; Rodríguez-Arribas, M.; Galindo, M.F.; Jordan, J.; Casado-Naranjo, I.; Fuentes, J.M.; Yakhine-Diop, S.M.S. Vitamin D Receptor Polymorphisms in a Spanish Cohort of Parkinson's Disease Patients. *Genet Test Mol Biomarkers* **2024**, *28*, 59–64, doi:10.1089/gtmb.2023.0344.

62. Kundu, N.C.; Kundu, A.; Khalil, M.I.; Joy, K.M.N.I.; Sen, M.; Hasan, Z.; Sahabuddin, M.; Rafi, M.A.; Hasan, M.J. A Case-Control Study on Vitamin D Receptor Gene Polymorphisms in Patients with Parkinson's Disease in Bangladesh. *Sci Rep* **2025**, *15*, 12333, doi:10.1038/s41598-025-96195-0.

63. Gröninger, M.; Sabin, J.; Kaaks, R.; Amiano, P.; Aune, D.; Castro, N.C.; Guevara, M.; Hansen, J.; Homann, J.; Masala, G.; et al. Associations of Milk, Dairy Products, Calcium and Vitamin D Intake with Risk of Developing Parkinson´s Disease within the EPIC4ND Cohort. *Eur J Epidemiol* **2024**, *39*, 1251–1265, doi:10.1007/s10654-024-01183-9.

64. Veronese, N.; Nova, A.; Fazia, T.; Riggi, E.; Yang, L.; Piccio, L.; Huang, B.-H.; Ahmadi, M.; Barbagallo, M.; Notarnicola, M.; et al. Contribution of Nutritional, Lifestyle, and Metabolic Risk Factors to Parkinson's Disease. *Mov Disord* **2024**, *39*, 1203–1212, doi:10.1002/mds.29778.

65. Furman, I.; Baudet, C.; Brachet, P. Differential Expression of M-CSF, LIF, and TNF-Alpha Genes in Normal and Malignant Rat Glial Cells: Regulation by Lipopolysaccharide and Vitamin D. *J Neurosci Res* **1996**, *46*, 360–366, doi:10.1002/(SICI)1097-4547(19961101)46:3%3C360::AID-JNR9%3E3.0.CO;2-I.

66. Puchacz, E.; Stumpf, W.E.; Stachowiak, E.K.; Stachowiak, M.K. Vitamin D Increases Expression of the Tyrosine Hydroxylase Gene in Adrenal Medullary Cells. *Brain Res Mol Brain Res* **1996**, *36*, 193–196, doi:10.1016/0169-328x(95)00314-i.

67. Musiol, I.M.; Feldman, D. 1,25-Dihydroxyvitamin D3 Induction of Nerve Growth Factor in L929 Mouse Fibroblasts: Effect of Vitamin D Receptor Regulation and Potency of Vitamin D3 Analogs. *Endocrinology* **1997**, *138*, 12–18, doi:10.1210/endo.138.1.4858.

68. Garcion, E.; Sindji, L.; Leblondel, G.; Brachet, P.; Darcy, F. 1,25-Dihydroxyvitamin D3 Regulates the Synthesis of Gamma-Glutamyl Transpeptidase and Glutathione Levels in Rat Primary Astrocytes. *J Neurochem* **1999**, *73*, 859–866, doi:10.1046/j.1471-4159.1999.0730859.x.

69. Shinpo, K.; Kikuchi, S.; Sasaki, H.; Moriwaka, F.; Tashiro, K. Effect of 1,25-Dihydroxyvitamin D(3) on Cultured Mesencephalic Dopaminergic Neurons to the Combined Toxicity Caused by L-Buthionine Sulfoximine and 1-Methyl-4-Phenylpyridine. *J Neurosci Res* **2000**, *62*, 374–382, doi:10.1002/1097-4547(20001101)62:3%3C374::AID-JNR7%3E3.0.CO;2-7.

70. Ibi, M.; Sawada, H.; Nakanishi, M.; Kume, T.; Katsuki, H.; Kaneko, S.; Shimohama, S.; Akaike, A. Protective Effects of 1 Alpha,25-(OH)(2)D(3) against the Neurotoxicity of Glutamate and Reactive Oxygen Species in Mesencephalic Culture. *Neuropharmacology* **2001**, *40*, 761–771, doi:10.1016/s0028-3908(01)00009-0.

71. Wang, J.Y.; Wu, J.N.; Cherng, T.L.; Hoffer, B.J.; Chen, H.H.; Borlongan, C.V.; Wang, Y. Vitamin D(3) Attenuates 6-Hydroxydopamine-Induced Neurotoxicity in Rats. *Brain Res* **2001**, *904*, 67–75, doi:10.1016/s0006-8993(01)02450-7.

72. Cui, X.; McGrath, J.J.; Burne, T.H.J.; Mackay-Sim, A.; Eyles, D.W. Maternal Vitamin D Depletion Alters Neurogenesis in the Developing Rat Brain. *Int J Dev Neurosci* **2007**, *25*, 227–232, doi:10.1016/j.ijdevneu.2007.03.006.

73. Orme, R.P.; Bhangal, M.S.; Fricker, R.A. Calcitriol Imparts Neuroprotection in Vitro to Midbrain Dopaminergic Neurons by Upregulating GDNF Expression. *PLoS One* **2013**, *8*, e62040, doi:10.1371/journal.pone.0062040.

74. Jang, W.; Kim, H.J.; Li, H.; Jo, K.D.; Lee, M.K.; Song, S.H.; Yang, H.O. 1,25-Dyhydroxyvitamin D_3 Attenuates Rotenone-Induced Neurotoxicity in SH-SY5Y Cells through Induction of Autophagy. *Biochem Biophys Res Commun* **2014**, *451*, 142–147, doi:10.1016/j.bbrc.2014.07.081.

75. Jang, W.; Park, H.-H.; Lee, K.-Y.; Lee, Y.J.; Kim, H.-T.; Koh, S.-H. 1,25-Dyhydroxyvitamin D3 Attenuates L-DOPA-Induced Neurotoxicity in Neural Stem Cells. *Mol Neurobiol* **2015**, *51*, 558–570, doi:10.1007/s12035-014-8835-1.

76. Pertile, R.A.N.; Cui, X.; Eyles, D.W. Vitamin D Signaling and the Differentiation of Developing Dopamine Systems. *Neuroscience* **2016**, *333*, 193–203, doi:10.1016/j.neuroscience.2016.07.020.

77. Cataldi, S.; Arcuri, C.; Hunot, S.; Légeron, F.-P.; Mecca, C.; Garcia-Gil, M.; Lazzarini, A.; Codini, M.; Beccari, T.; Tasegian, A.; et al. Neutral Sphingomyelinase Behaviour in Hippocampus Neuroinflammation of MPTP-Induced Mouse Model of Parkinson's Disease and in Embryonic Hippocampal Cells. *Mediators Inflamm* **2017**, *2017*, 2470950, doi:10.1155/2017/2470950.

78. Rcom-H'cheo-Gauthier, A.N.; Meedeniya, A.C.B.; Pountney, D.L. Calcipotriol Inhibits α-Synuclein Aggregation in SH-SY5Y Neuroblastoma Cells by a Calbindin-D28k-Dependent Mechanism. *J Neurochem* **2017**, *141*, 263–274, doi:10.1111/jnc.13971.

79. Cataldi, S.; Arcuri, C.; Hunot, S.; Mecca, C.; Codini, M.; Laurenti, M.E.; Ferri, I.; Loreti, E.; Garcia-Gil, M.; Traina, G.; et al. Effect of Vitamin D in HN9.10e Embryonic Hippocampal Cells and in Hippocampus from MPTP-Induced Parkinson's Disease Mouse Model. *Front Cell Neurosci* **2018**, *12*, 31, doi:10.3389/fncel.2018.00031.

80. Pertile, R.A.N.; Cui, X.; Hammond, L.; Eyles, D.W. Vitamin D Regulation of GDNF/Ret Signaling in Dopaminergic Neurons. *FASEB J* **2018**, *32*, 819–828, doi:10.1096/fj.201700713R.

81. Hu, J.; Wu, J.; Wan, F.; Kou, L.; Yin, S.; Sun, Y.; Li, Y.; Zhou, Q.; Wang, T. Calcitriol Alleviates MPP(+)- and MPTP-Induced Parthanatos Through the VDR/PARP1 Pathway in the Model of Parkinson's Disease. *Front Aging Neurosci* **2021**, *13*, 657095, doi:10.3389/fnagi.2021.657095.

82. Zhang, Y.; Ji, W.; Zhang, S.; Gao, N.; Xu, T.; Wang, X.; Zhang, M. Vitamin D Inhibits the Early Aggregation of α-Synuclein and Modulates Exocytosis Revealed by Electrochemical Measurements. *Angew Chem Int Ed Engl* **2022**, *61*, e202111853, doi:10.1002/anie.202111853.

83. de Siqueira, E.A.; Magalhães, E.P.; de Assis, A.L.C.; Sampaio, T.L.; Lima, D.B.; Marinho, M.M.; Martins, A.M.C.; de Andrade, G.M.; de Barros Viana, G.S. 1α,25-Dihydroxyvitamin D3 (VD3) Shows a Neuroprotective Action Against Rotenone Toxicity on PC12 Cells: An In Vitro Model of Parkinson's Disease. *Neurochem Res* **2023**, *48*, 250–262, doi:10.1007/s11064-022-03735-5.

84. Sanchez, B.; Lopez-Martin, E.; Segura, C.; Labandeira-Garcia, J.L.; Perez-Fernandez, R. 1,25-Dihydroxyvitamin D(3) Increases Striatal GDNF mRNA and Protein Expression in Adult Rats. *Brain Res Mol Brain Res* **2002**, *108*, 143–146, doi:10.1016/s0169-328x(02)00545-4.

85. Lin, A.M.Y.; Fan, S.F.; Yang, D.M.; Hsu, L.L.; Yang, C.H.J. Zinc-Induced Apoptosis in Substantia Nigra of Rat Brain: Neuroprotection by Vitamin D3. *Free Radic Biol Med* **2003**, *34*, 1416–1425, doi:10.1016/s0891-5849(03)00105-9.

86. Kalueff, A.V.; Lou, Y.-R.; Laaksi, I.; Tuohimaa, P. Impaired Motor Performance in Mice Lacking Neurosteroid Vitamin D Receptors. *Brain Res Bull* **2004**, *64*, 25–29, doi:10.1016/j.brainresbull.2004.04.015.

87. Burne, T.H.J.; McGrath, J.J.; Eyles, D.W.; Mackay-Sim, A. Behavioural Characterization of Vitamin D Receptor Knockout Mice. *Behav Brain Res* **2005**, *157*, 299–308, doi:10.1016/j.bbr.2004.07.008.

88. Kim, J.-S.; Ryu, S.-Y.; Yun, I.; Kim, W.-J.; Lee, K.-S.; Park, J.-W.; Kim, Y.-I. 1alpha,25-Dihydroxyvitamin D(3) Protects Dopaminergic Neurons in Rodent Models of Parkinson's Disease through Inhibition of Microglial Activation. *J Clin Neurol* **2006**, *2*, 252–257, doi:10.3988/jcn.2006.2.4.252.

89. Smith, M.P.; Fletcher-Turner, A.; Yurek, D.M.; Cass, W.A. Calcitriol Protection against Dopamine Loss Induced by Intracerebroventricular Administration of 6-Hydroxydopamine. *Neurochem Res* **2006**, *31*, 533–539, doi:10.1007/s11064-006-9048-4.

90. Sanchez, B.; Relova, J.L.; Gallego, R.; Ben-Batalla, I.; Perez-Fernandez, R. 1,25-Dihydroxyvitamin D3 Administration to 6-Hydroxydopamine-Lesioned Rats Increases Glial Cell Line-Derived Neurotrophic Factor and Partially Restores Tyrosine Hydroxylase Expression in Substantia Nigra and Striatum. *J Neurosci Res* **2009**, *87*, 723–732, doi:10.1002/jnr.21878.

91. Cui, X.; Pelekanos, M.; Burne, T.H.J.; McGrath, J.J.; Eyles, D.W. Maternal Vitamin D Deficiency Alters the Expression of Genes Involved in Dopamine Specification in the Developing Rat Mesencephalon. *Neurosci Lett* **2010**, *486*, 220–223, doi:10.1016/j.neulet.2010.09.057.

92. Cass, W.A.; Peters, L.E.; Fletcher, A.M.; Yurek, D.M. Evoked Dopamine Overflow Is Augmented in the Striatum of Calcitriol Treated Rats. *Neurochem Int* **2012**, *60*, 186–191, doi:10.1016/j.neuint.2011.11.010.

93. Dean, E.D.; Mexas, L.M.; Cápiro, N.L.; McKeon, J.E.; DeLong, M.R.; Pennell, K.D.; Doorn, J.A.; Tangpricha, V.; Miller, G.W.; Evatt, M.L. 25-Hydroxyvitamin D Depletion Does Not Exacerbate MPTP-Induced Dopamine Neuron Damage in Mice. *PLoS One* **2012**, *7*, e39227, doi:10.1371/journal.pone.0039227.

94. Hashemvarzi, S.A.; Samadi, B.; Khazaeli, N. Preconditioning Effect of Aerobic Exercise with Vitamin D3 Intake on VEGF Levels in 6-OHDA-Lesioned Rat Model of Parkinson's Disease. *Journal of Basic and Clinical Pathophysiology* **2017**, *5*, 1–8.

95. Jiang, P.; Zhang, W.-Y.; Li, H.-D.; Cai, H.-L.; Xue, Y. Repeated Haloperidol Administration Has No Effect on Vitamin D Signaling but Increase Retinoid X Receptors and Nur77 Expression in Rat Prefrontal Cortex. *Cell Mol Neurobiol* **2013**, *33*, 309–312, doi:10.1007/s10571-012-9902-7.

96. Cass, W.A.; Peters, L.E.; Fletcher, A.M.; Yurek, D.M. Calcitriol Promotes Augmented Dopamine Release in the Lesioned Striatum of 6-Hydroxydopamine Treated Rats. *Neurochem Res* **2014**, *39*, 1467–1476, doi:10.1007/s11064-014-1331-1.

97. Li, H.; Jang, W.; Kim, H.J.; Jo, K.D.; Lee, M.K.; Song, S.H.; Yang, H.O. Biochemical Protective Effect of 1,25-Dihydroxyvitamin D3 through Autophagy Induction in the MPTP Mouse Model of Parkinson's Disease. *Neuroreport* **2015**, *26*, 669–674, doi:10.1097/WNR.0000000000000401.

98. Mark, K.A.; Dumas, K.J.; Bhaumik, D.; Schilling, B.; Davis, S.; Oron, T.R.; Sorensen, D.J.; Lucanic, M.; Brem, R.B.; Melov, S.; et al. Vitamin D Promotes Protein Homeostasis and Longevity via the Stress Response Pathway Genes Skn-1, Ire-1, and Xbp-1. *Cell Rep* **2016**, *17*, 1227–1237, doi:10.1016/j.celrep.2016.09.086.

99. Calvello, R.; Cianciulli, A.; Nicolardi, G.; De Nuccio, F.; Giannotti, L.; Salvatore, R.; Porro, C.; Trotta, T.; Panaro, M.A.; Lofrumento, D.D. Vitamin D Treatment Attenuates Neuroinflammation and Dopaminergic Neurodegeneration in an Animal Model of Parkinson's Disease, Shifting M1 to M2 Microglia Responses. *J Neuroimmune Pharmacol* **2017**, *12*, 327–339, doi:10.1007/s11481-016-9720-7.

100. Cass, W.A.; Peters, L.E. Reduced Ability of Calcitriol to Promote Augmented Dopamine Release in the Lesioned Striatum of Aged Rats. *Neurochem Int* **2017**, *108*, 222–229, doi:10.1016/j.neuint.2017.04.001.

101. Lima, L.A.R.; Lopes, M.J.P.; Costa, R.O.; Lima, F.A.V.; Neves, K.R.T.; Calou, I.B.F.; Andrade, G.M.; Viana, G.S.B. Vitamin D Protects Dopaminergic Neurons against Neuroinflammation and Oxidative Stress in Hemiparkinsonian Rats. *J Neuroinflammation* **2018**, *15*, 249, doi:10.1186/s12974-018-1266-6.

102. Kim, H.; Shin, J.-Y.; Lee, Y.-S.; Yun, S.P.; Maeng, H.-J.; Lee, Y. Brain Endothelial P-Glycoprotein Level Is Reduced in Parkinson's Disease via a Vitamin D Receptor-Dependent Pathway. *Int J Mol Sci* **2020**, *21*, doi:10.3390/ijms21228538.

103. Ding, M.-Y.; Peng, Y.; Li, F.; Li, Z.-Q.; Wang, D.; Zhou, G.-C.; Wang, Y. Andrographolide Derivative as Antagonist of Vitamin D Receptor to Induce Lipidation of Microtubule Associate Protein 1 Light Chain 3 (LC3). *Bioorg Med Chem* **2021**, *51*, 116505, doi:10.1016/j.bmc.2021.116505.

104. Araújo de Lima, L.; Oliveira Cunha, P.L.; Felicio Calou, I.B.; Tavares Neves, K.R.; Facundo, H.T.; Socorro de Barros Viana, G. Effects of Vitamin D (VD3) Supplementation on the Brain Mitochondrial Function of Male Rats, in the 6-OHDA-Induced Model of Parkinson's Disease. *Neurochem Int* **2022**, *154*, 105280, doi:10.1016/j.neuint.2022.105280.

105. Bayo-Olugbami, A.; Nafiu, A.B.; Amin, A.; Ogundele, O.M.; Lee, C.C.; Owoyele, B.V. Vitamin D Attenuated 6-OHDA-Induced Behavioural Deficits, Dopamine Dysmetabolism, Oxidative Stress, and Neuro-Inflammation in Mice. *Nutr Neurosci* **2022**, *25*, 823–834, doi:10.1080/1028415X.2020.1815331.

106. Magdy, A.; Farrag, E.A.E.; Hamed, S.M.; Abdallah, Z.; El Nashar, E.M.; Alghamdi, M.A.; Ali, A.A.H.; Abd El-Kader, M. Neuroprotective and Therapeutic Effects of Calcitriol in Rotenone-Induced Parkinson's Disease Rat Model. *Front Cell Neurosci* **2022**, *16*, 967813, doi:10.3389/fncel.2022.967813.

107. da Costa, R.O.; Gadelha-Filho, C.V.J.; de Aquino, P.E.A.; Lima, L.A.R.; de Lucena, J.D.; Ribeiro, W.L.C.; Lima, F.A.V.; Neves, K.R.T.; de Barros Viana, G.S. Vitamin D (VD3) Intensifies the Effects of Exercise and Prevents Alterations of Behavior, Brain Oxidative Stress, and Neuroinflammation, in Hemiparkinsonian Rats. *Neurochem Res* **2023**, *48*, 142–160, doi:10.1007/s11064-022-03728-4.

108. Imam, R.A.; Abdel-Hamed, M.R. Vitamin D3 Promotes Oligodendrogenesis and Modulates Synucleinopathy in Lead-Induced Nigral Pars Compacta Neurotoxicity in Rats. *Folia Morphol (Warsz)* **2023**, *82*, 42–52, doi:10.5603/FM.a2022.0003.

109. Khosravi, F.; Mirzaei, S.; Hojati, V.; Hashemi, M.; Entezari, M. Co-Administration of Vitamins B12 and D During Pregnancy Have Strong Neuroprotective Effects in Parkinson Disease. *Mol Neurobiol* **2023**, *60*, 1986–1996, doi:10.1007/s12035-022-03186-7.

https://doi.org/10.3390/neurosci6040130
